# Dual-Drug Delivery by Anisotropic and Uniform Hybrid Nanostructures: A Comparative Study of the Function and Substrate–Drug Interaction Properties

**DOI:** 10.3390/pharmaceutics15041214

**Published:** 2023-04-11

**Authors:** Delaram Kargari Aghmiouni, Sepideh Khoee

**Affiliations:** Polymer Laboratory, School of Chemistry, College of Science, University of Tehran, Tehran 14155-6455, Iran

**Keywords:** co-delivery systems, dual-drug delivery, homogeneous nanoparticles, anisotropic nanoparticles, Janus nanoparticles

## Abstract

By utilizing nanoparticles to upload and interact with several pharmaceuticals in varying methods, the primary obstacles associated with loading two or more medications or cargos with different characteristics may be addressed. Therefore, it is feasible to evaluate the benefits provided by co-delivery systems utilizing nanoparticles by investigating the properties and functions of the commonly used structures, such as multi- or simultaneous-stage controlled release, synergic effect, enhanced targetability, and internalization. However, due to the unique surface or core features of each hybrid design, the eventual drug–carrier interactions, release, and penetration processes may vary. Our review article focused on the drug’s loading, binding interactions, release, physiochemical, and surface functionalization features, as well as the varying internalization and cytotoxicity of each structure that may aid in the selection of an appropriate design. This was achieved by comparing the actions of uniform-surfaced hybrid particles (such as core–shell particles) to those of anisotropic, asymmetrical hybrid particles (such as Janus, multicompartment, or patchy particles). Information is provided on the use of homogeneous or heterogeneous particles with specified characteristics for the simultaneous delivery of various cargos, possibly enhancing the efficacy of treatment techniques for illnesses such as cancer.

## 1. Introduction

The usage of nanoparticles as potential carriers for drugs and therapeutic cargos has been investigated widely by many research studies. FDA-approved nanoparticles, including inorganic materials such as porous silica [1], metal oxides [2], metals such as silver, gold [3,4], and synthetic or natural polymers [5], have been widely employed for improved drug delivery systems. Reducing bio-distribution, allowing for gradual and steady drug release, protecting pharmaceuticals from degradation, and extending their half-life are just a few of the ways in which nanoparticles, as drug delivery agents, excel above “free” drugs. Drugs with a low therapeutic index, hydrophobic and poorly water soluble and quickly degrading nature, becoming unstable, accumulating in undesirable tissues and inducing severe toxicity, etc., can all be used in the medical sector owing to nanoparticles [6]. In recent years, nanoparticulated vehicles that can transport multiple medications in a controlled manner have been the subject of many studies due to the success of therapies that accurately combine pharmaceuticals to enhance their synergistic effect [7]. As part of the co-delivery approach, both the administration of two anticancer drugs concurrently and the release of an anticancer agent in combination with a therapeutic molecule with anti-inflammatory, immunologic, antitumor, or anti-angiogenic capabilities were explored [8]. Co-delivery of therapeutic molecules using nanoparticles displays many advantages over the delivery of a single molecule or drug. One of the most significant benefits of co-delivery is the synergistic effect, in which two drugs may operate on different routes simultaneously, improving the killing rate of tumor cells and preventing the occurrence of multidrug resistance (MDR) [9]. Other benefits brought by co-delivery utilizing nanoparticles include increased absorption by cells [10], reduced side effects owing to specific selectivity [11], increased cytotoxicity [12], and solubility [13] of the medications.

However, crucial factors determining the eventual function of pharmaceuticals carried via nanoparticles in vivo, not only for the single molecule/drug but also for the co-delivery systems, are size, shape, composition, surface structure, and charge [14,15,16,17,18,19]. The process of pharmaceutical loading and release is also intricately intertwined with interactions. For instance, hydrophobic interaction, electrostatic attraction, hydrogen bonding, and π–π stacking are all examples of drug–carrier interactions that can affect the loading mechanism and efficiency in post-loading strategies, in which nanoparticles are synthesized first, and the loading of cargo is carried out in a later step [20]. In addition, physicochemical properties, such as surface chemistry, can control the interaction of nanoparticles (NPs) with plasma proteins, such as immune proteins [21], the permeability of cell membranes, the rate of cell absorption, the specificity with which they identify targets, and the stimulation of cellular uptake [22,23]. Therefore, it is important to consider the influence of the aforementioned parameters on the in vivo operations, especially in terms of co-loaded molecules in a single carrier that may exhibit undesirable or desirable phenomena such as drug–drug interactions [24]. Therefore, investigating these parameters, such as interactions, composition, surface, and core structure, may clarify their effect on co-delivery systems’ performances. Accordingly, nanoparticles’ surface structures might vary depending on their manufacturing process and final application. For instance, different synthesis methods have been employed to create various nanoparticles with uniform or isotropic surface structures, such as lipid-based NPs, micelles, polymeric nanoparticles, and inorganic/metallic nanoparticles, where the cargos may be loaded in any storage space from surface to core [25,26,27,28]. Different types of anisotropic nanoparticles, such as Janus, multicompartment, and patchy particles, may be created via various methods. In contrast to Janus NPs, which have two discrete phases separated in the corona and the core, patchy NPs contain domains with different compositions on their surface, while multicompartment particles possess them in their core [29].

The disrupted symmetry of these anisotropic nanoparticles, particularly Janus nanoparticles, provides efficient paths for the incorporation of properties hitherto unknown. Janus nanoparticles with two-faced structures enable the incorporation of two chemically incompatible substances into a single particle with interesting amphiphilic, optical, magnetic, and catalytic properties [30]. In contrast to homogenous or core–shell nanoparticles, which usually contain one kind of surface (hydrophobic or hydrophilic), amphiphilic Janus nanoparticles include both types of surfaces inside one nanoparticle and have a longer blood circulation time [31]. In addition, Janus nanoparticles exhibit the optimal qualities for dual hydrophilic and hydrophobic drug delivery when their separate areas are modified with hydrophilic and hydrophobic features [32]. However, there are also challenges regarding these anisotropic structures, such as the difficulty of synthesis due to their heterogeneous structures, the range of utilized material from inorganic to organic polymers approved by the FDA, and the widely explored proof-of-concept research without the actual varied clinical research [33]. Moreover, the number of studies on the application of these nanoparticles, especially Janus NPs, in co-delivery systems and with the purpose of synergic effect investigation is much fewer than isotropic/uniform NPs. 

Overall, according to the advantages and drawbacks of both uniform and anisotropic structures, the selection of the appropriate substrate depends on the objective of employing the simultaneous drug delivery system. In this review, the performance of nanoparticles in two categories, uniform and anisotropic, is analyzed, where the critical aspects of solubility of pharmaceuticals employing substrate, encapsulation data, interactions and release mechanism, uptake and cytotoxicity have been studied in various papers.

**Scheme 1:**
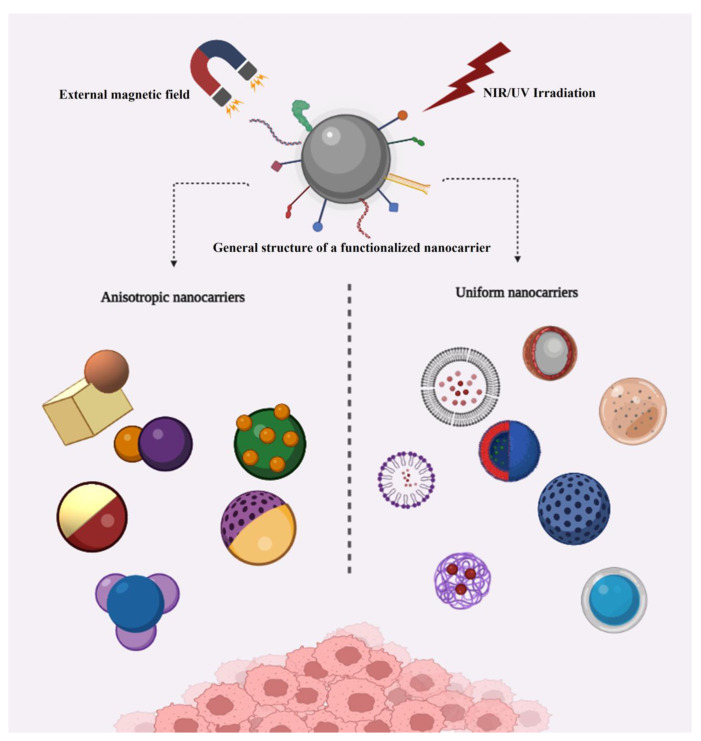
A graphical overview of the types of nanoparticles in uniform and anisotropic structures suitable for usage in dual-drug delivery systems. Created with Biorender.com.

In this manner, by studying the most essential functional features of dual/co-delivery, crucial information is gained about the influence of the type of structure on the final usage of pharmaceuticals, which contributes to a better and more appropriate selection of substrate or combination of drugs (Scheme 1).

## 2. Uniform Structures

Structures with a uniform surface are frequently used in drug delivery systems that transport several medicines or genes [26]. The outer layer of these invariant structures is fabricated from a single component that may be coated onto a polymeric, mineral, or metallic-based core to take advantage of the hybrid structures’ various properties. In addition, the surface structure permits the attachment of ligands, proteins, or medical cargos by the use of diverse surface functional groups and linkers. Moreover, a limited fraction of the medicine could be stored in the core of core–shell complexes. In micelles, for instance, two kinds of pharmaceuticals with different solubilities can collect in the outer layers and the core, allowing for the simultaneous delivery of their payloads. Important aspects of drug delivery systems are exemplified by the following instances of uniform nanoparticles, demonstrating the efficiency of their application in delivering two or more pharmaceuticals.

### 2.1. Solubility and Encapsulation

One of the main factors in achieving the ideal pharmacological effect of a drug is its solubility in water. Any drug needs to be in an aqueous medium at the absorption site [34]. Poorly soluble compounds account for an estimated 40% of already authorized medications and approximately 90% of drugs in the production phase [35]. Therefore, various techniques such as pH modification [36], co-solvency [37], size reduction [38], self-emulsifying [39], usage of liposomes [40], polymers [41], and nanoparticles [42] have been designed to improve the solubility of the poorly soluble drugs. Inadequate solubilization, higher critical micellar concentrations (CMC), and probable side effects following intravenous injection prevented the widespread use of hydrophilic surfactants formerly used to solubilize the drug for oral and intravenous administration. However, in contrast to other surfactants, polymeric micelles, specifically those containing polyethylene glycol (PEG) as one of their block components, have advantages in encapsulation, solubility, tolerability, and longer circulation times owing to their low CMC [43]. Functionalization of nanoparticles or drugs with PEG permits the better conjugation of medications to the particles, so enhancing their solubility and bioavailability, and also providing a stealthier delivery mechanism [44,45,46]. For instance, in a single-drug loading system, curcumin, a poorly soluble drug in water, has been conjugated with PEG and by using EDS/NHS linkers, was connected to the surface of magnetic nanoparticles in order to overcome its hydrophobic properties and take advantage of the magnetic property of nanocarriers. According to their release profiles, at a certain time, the release rate of free curcumin is lower than that of nanoparticles containing curcumin, indicating that the nanoformulation could increase the solubility of curcumin. An improved hydrolysis rate of the curcumin–PEG bond at an acidic pH compared to a neutral pH also confirmed the enhanced solubility of curcumin [47]. 

However, in dual delivery platforms, the difficulty lies in delivering several compounds to the targeted site using a single-drug delivery system, as is crucial for combined therapy [48,49] due to their various physiochemical features [50]. However, the type of carriers and their synthesis technique may address the challenges of dual encapsulation. For example, the biocompatible and biodegradable poly(D,L-lactic-co-glycolic acid) PLGA was used as a potential carrier for two drugs, with the help of their drug release mechanisms through cleavage of the ester groups in their structure [51,52]. The co-encapsulation of the two anti-inflammatory painkillers diclofenac sodium (DS) and dexamethasone (DX), which possess different polarities (hydrophilic and hydrophobic, respectively), was performed using an o/w single-emulsion solvent-evaporation method, with a modification of using two miscible solvents (ethyl acetate: methanol 9:1 *v*/*v*) to increase the encapsulation rate and synergic effect of drugs with differing physiochemical properties. Consequently, the design of the nearly 140–160 nm polymeric particles was successful in increasing the uptake of the two drugs by utilizing dual solvent systems which also increased the solubility of the drugs in the organic medium in the optimal formulation of the drug: polymer 1:10, and 5% *w*/*v* of surfactant concentration [53]. 

In more recent research, graphene oxide sheets (GO) were used as a base carrier for the simultaneous delivery of DOX and curcumin, where DOX, as a widely used anticancer drug, is water soluble, whereas, curcumin has hydrophobic nature and poor bioavailability [19,54,55]. GO, to which the chemicals can be attached via π–π stacking, hydrophobic or electrostatic interactions, and covalent bonds was further functionalized at the edges with dendric poly(epichlorohydrin)-*graft*-hyperbranched polyglycerol (PCH-*g*-HPG) to improve solubility and hydrophilicity as the drug carrier, allowing for greater exploitation of the synergistic effect of the two drugs. A significant decrease in the solubility of nanocarriers was due to the replacement of chloride groups in the main chain of polymer with hydrazine with further modification by PCH. DOX was either covalently linked to the hydrazine groups or trapped in the polymer’s pores, while the loading mechanism of curcumin was through the π–π stacking interactions. The release of the two drugs in PBS at pHs 5 and 7.4 exhibited higher release rates when compared to the release of the single drug from the carriers, indicating an increase in bioavailability and solubility in aqueous media utilizing an oxygen-rich polymer modified GO carrier [56]. 

A similar combination of drugs, i.e., DOX and curcumin, was the subject of another research project in which the drugs were encapsulated in an amphiphilic polypeptide material mPEG-b-P(Glu-co-Phe), which forms a nanoparticle in water. The final nanoparticles demonstrated hydrophilicity and as well as hydrophobic stability owing to benzene groups in the structure. Comparing the encapsulation of the two drugs to their single conjugation with the co–polymer, it was discovered that the presence of DOX formed a hydrophobic core, enhancing the loading rate of the CUR to 8.1% from 1.14% in the single-drug assessment, suggesting improved encapsulation and solubility of the CUR when embedded in the carrier core combined with the DOX with a marginally lower encapsulation rate in the dual delivery system [57]. 

In a study in 2017 by Medel et al., hydrophobic water-insoluble anticancer drugs curcumin (CUR) and bortezomib (BTZ) were loaded in a diblock copolymer methoxy-poly (ethylene glycol)-*block*-poly lactic acid (mPEG-*b*-PLA) to assess their loading, cellular uptake by HeLa, MCF-7, and MDA-MB-231 cells’ efficiencies. Bortezomib, a highly selective 26S proteasome inhibitor, has been used successfully in recent years to treat patients with multiple myeloma; and its efficacy in breast cancer treatment has been the subject of several studies [58,59,60]. The combination of these drugs forms a stable boronate ester compound at neutral or alkaline pHs that decomposes in acidic environments (such as endosomes and tumor sites) and contributes to their release and antitumor synergy. Moreover, the drug loading content and efficiency assessments indicate a 2.5% and 20–25% rate, respectively. Depending on the copolymer structure, an increase in the length of the hydrophobic section of the copolymer enhanced the drug loading amount slightly. Curcumin’s efficiency also rises in the form of complex, as well as its water solubility from 11 to 30,000 mg/mL [61]. 

Inorganic porous materials, namely mesoporous silica nanoparticles (MSNs) [62] and carbons [63], metal-organic frameworks (MOFs) [64], exhibited superior mechanical and chemical stability under bodily conditions, control of the diffusion and release rate of the encapsulated compounds, in comparison to other kinds of materials [65]. Importantly, their porous nature may aid in the controlled diffusion and solubility enhancement of poorly soluble pharmaceuticals [66]. Mesoporous silica nanoparticles, in particular, have been studied widely due to their wide application in drug delivery systems with different approaches including targeted, controlled, and sustained delivery [67]. Owing to their structure rich in functional groups, successful conjugation with various pharmaceuticals (APIs) has made them widely used to deliver drugs specifically in the treatment of cancer [67,68,69,70]. 

The mesoporous silica nanoparticle in a project was designed as the carrier for the chemotherapy treatments using a combination of drugs, doxorubicin and camptothecin (CPT). The Chinese tree Camptotheca acuminate was the source of CPT, a topoisomerase-1 inhibitor that has an anticancer effect by binding to the topoisomerase-1 and DNA complex [71,72]. However, CPT’s therapeutic use is constrained by its poor water solubility and unpredictable potency [73]. This drawback affects not just treatments but also drug loading and release from DDSs. Consequently, a prodrug with more amphiphilicity than CPT but with the same cytotoxicity was considered to overcome these problems. PEGylation was the technique for increasing the solubility of the hydrophobic drug CPT. Nevertheless, the introducing of a PEG chain may inhibit the biological effect of CPT. Therefore, a cleavable glutathione-sensitive linker is used for attaching the PEG chain to the drug. Following the synthesis of MSNs and loading of the CPT-PEG prodrug into their pores, Dihydrazide polyethylene glycol chains will be added to the surface of aldehyde-functionalized MSNs to cover voids using hydrazone linkages. The second dihydrazide group will react with the DOX structure’s ketone group. The better loading rates of the CPT-PEG compared to CPT indicate the improvement of the hydrophobic drug’s solubility, in addition to the better cytotoxicity of HepG2 cells using the combination of DOX and CPT-PEG [74].

Chemotherapy for tumors can be hampered by a phenomenon called multidrug resistance (MDR). MDR refers to a pattern of resistance in which tumor cells show resistance to many medications with no structural similarities and diverse molecular targets [75]. A combination of poor drug delivery and epigenetic and genetic abnormalities in antibiotic susceptibility toward the cancer cell promotes multidrug resistance [76]. Co-delivery of antitumor Paclitaxel (PTX) and MDR reversal agent tetrandrine (TET) was established by employing mesoporous silica nanoparticles as carriers in order to defeat the MDR of MCF-7/ADR cells. Paclitaxel is extensively used in clinical chemotherapy as a member of the anti-microtubule drug class for the treatment of cancer and as a typical component [77]. In many therapeutic studies and clinical trials, paclitaxel was found to be significantly effective in breast cancer [78]. Tetrandrine (TET) is a relatively non-toxic medication with a significant reversal impact in vitro and in vivo on P-gp-mediated MDR [79] where through its role as a mediator of ATP-dependent efflux, p-gp protein layer facilitates the removal of drugs, contributing to MDR [80]. The use of MSNs as their drug carrier with the beneficial properties of the silica nanoparticles, such as biocompatibility and tunable pore size/particle size, is considered to take advantage of the combination of drugs and increase the antitumor effect and intercellular concentration of PTX. Accordingly, MSNs are considered a type of organic–inorganic composite before the surfactant templates’ removal. Solubilization of PTX and TET in surfactant micelles allows for concurrent hydrolysis and condensation of silica resources to produce nanoparticles, as both compounds are insoluble in water. Nanoparticles encasing drug-containing hexadecyltrimethylammonium bromide (CTAB) were formed by a self-assembly drug-loading method, including the solubilization of pharmaceuticals in the CTAB core as the surfactant and the hydrolysis and condensation of TEOS as silica precursor. Moreover, XRD analysis of the particles displayed no crystalline peaks for pure drugs or CTAB, which may be explained by the solubilization of both medications inside the hydrophobic core of the CTAB in their molecular amphoteric form. The micelles and drugs are then enclosed in the nano-sized pores of the silica, where they are unable to form a crystalline structure due to the lack of space. Additionally, more hydrophobicity of PTX helped with its higher loading rate inside the core of CTAB micelles, lowering the size of the single-drug platform PTX-CTAB@MSN compared to TET-CTAB@MSN [81]. 

### 2.2. Release and Interactions

When it comes to the application of nanoparticles, drug release behavior is essential, as it affects formulation, drug stability, and therapeutic efficacy [82,83]. Desorption of the surface-bound or adsorbed drug, diffusion away from the polymer nanoparticles, erosion of the nanoparticles, and a combination of erosion and diffusion processes all play a role in the rates at which drugs are released from polymer nanoparticles. As a result, drug release is controlled by factors including diffusion and biodegradation. Rapid drug release from polymer nanoparticles, often known as “burst release”, is typically first seen. It has been observed that different types of drug delivery systems lead to different drug release characteristics [84]. Therefore, simultaneous analysis of the release profile and the interactions of the drug delivery system may help with the better choice of carrier and drugs.

**Figure 1:**
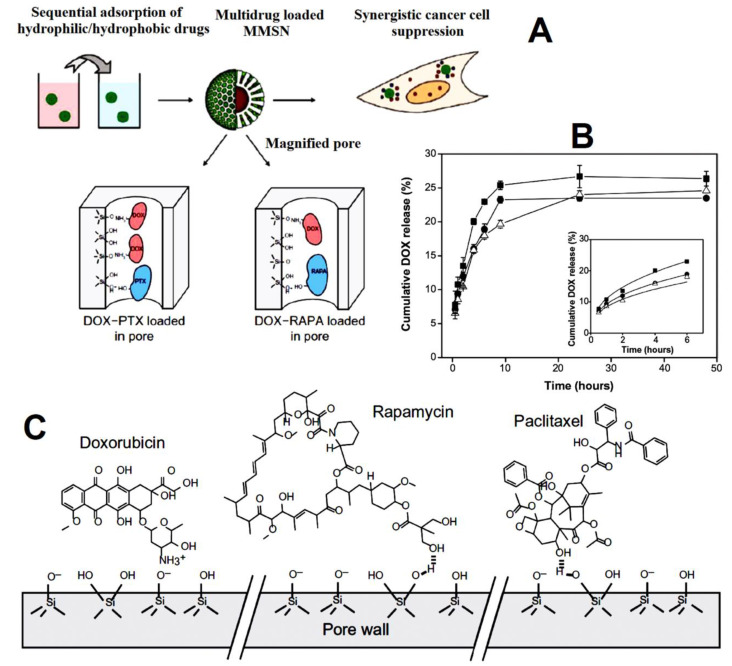
Release and interaction data of MMSN in co-delivery of DOX, RAPA, and PTX. (**A**) Synthesis design of MMSNs and loading interaction of drugs inside the pores. (**B**) DOX release rate individually (■), from DOX-PTX co-loaded NPs (●), from DOX-RAPA co-loaded NPs (∆). (**C**) The interactions of the mesoporous silica pore walls with drug molecules. Reproduced from Liu et al. [3], International Journal of Nanomedicine 2012 7 999-1013’ Originally published by and used with permission from Dove Medical Press Ltd.

In a 2012 study by Liu et al., magnetic mesoporous silica nanoparticles were synthesized as the structure for co-loading of hydrophilic DOX and hydrophobic drugs PTX and rapamycin (RAPA) in the tumor treatment analysis of A549 human pulmonary adenocarcinoma cells. Using the immunosuppressive medication rapamycin, it is feasible to prevent organ rejection following transplantation. In addition, it has a substantial cytostatic effect, as shown by its ability to inhibit the growth of human carcinoma cell lines from the NCI60 panel [85,86]. A comprehensive understanding of the interactions between the drugs and the MSN nanoparticles is feasible using the initial co-loading of the drugs. A sequential adsorption approach was used to load DOX from an aqueous solution and RAPA (or PTX) from the nonaqueous media into MMSNs, which suggest that by using two solvents, the appropriate ratios of the drugs are achievable. On the inner and outer pore surface of MCM-41 type mesoporous silica materials, accessible adsorption sites are composed of Q_2_ silanols [SiO]_2_Si[OH]_2_ (pKa~8.5) and Q_3_ silanols [SiO]_3_SiOH (pKa~2). Doxorubicin (DOX), as the hydrophilic compound (pKa 8.3), is positively charged in water and electrostatically adsorbed to the Q_3_ silanols of the negatively charged MMSNs while hydrogen bonds or polar interactions attract Q_2_ and Q_3_ silanols to the polar groups of the hydrophobic drugs in a nonaqueous medium (Figure 1). Through the sequential co-loading, it was observed that, when the amount of preloaded DOX was increased, the loading rates of all hydrophobic drugs decreased, explained by the fact that the surface-adsorbed DOX inhibits the absorption of hydrophobic drugs into pores by steric hindrance. Therefore, hydrophobic molecules have a somewhat unrestricted capacity to diffuse and transit through the pore channels in search of vacant spaces, which may be already unoccupied by the adsorbed DOX molecules at low DOX amounts.

The release study, in contrast, was only conducted for the DOX due to the fact that the release rates of the hydrophobic drugs in aqueous medium were negligible. The cumulative results of the three loading types, DOX-MMSN, DOX-RAPA-MMSN, and DOX-PTX-MMSN, were reported to have a burst release in the first 6 h, with subsequent releases reaching 26% for DOX-MMSN and 23% and 19% for DOX-PTX-MMSN and DOX-RAPA-MMSN, respectively. The Korsmeyer–Peppas model employed in the study for analyzing the release kinetics proposes that there are two stages of drug release: initially, the diffusion of the solvent into the pores to dissolve the substances, and subsequently, the release of the dissolved drugs from the pores, which results in a delayed total release from dual delivery systems. Therefore, the gradual release of the hydrophilic DOX may be justifiable because the pores become more hydrophobic in nature after loading with hydrophobic drugs, thus limiting the rapid release of DOX [87]. 

In a study on porous silicon material, an anti-inflammatory hydrophobic drug indomethacin (IMC) and peptide tyrosine tyrosine 3-36 (PYY3-36) were loaded in the two types of the Psi with different surface characteristics, thermally hydrocarbonized-Psi (THCPSi) and thermally oxidized-PSi (TOPSi). Indomethacin is a water-insoluble anti-inflammatory drug that has already been studied in the treatment of cancer [88,89,90]. Peptide YY (PYY) is a naturally produced peptide that belongs to the same family as neuropeptide Y (NPY) and pancreatic polypeptide (PP). PYY is a 36-amino acid gut peptide that is released in response to an increase in dietary energy intake by enteroendocrine L-cells in the digestive tract [91]. Different surface characteristics and zeta potentials, with −35.6 ± 0.9 mV for hydrophilic TOPSi and −37.1 ± 2.7 mV for hydrophobic THCPSi affecting loading rates via various electrostatic and hydrophobic interactions with drugs, are responsible for the observed discrepancy in drug adsorption. Due to incomplete IMC loading into the pores, where PYY3-36 is believed to be absorbed on the exterior surface of the particles via hydrophobic and electrostatic interactions, the dual-drug systems displayed higher loading rates than the single drug in both Psi types. Reductions in pH from 7.4 to 5.5 slowed the pace at which TOPSi and THCPSi NPs released IMC from either a single drug or a combination of drugs loaded into the NPs. Because of its low pKa of 4.5, IMC almost completely dissociated at pH 7.4, whereas only 90% was released at pH 5.5. This suggests that the quicker release rate of IMC at pH 7.4 may be due to the marginally higher solubility of IMC at pH 7.4 compared to that at pH 5.5. Similarly, PYY3-36 was released at a slower rate at pH 5.5 compared to neutral pH 7.4, the difference being attributed to the stronger attraction of the positively charged PYY3-36 in an acidic environment to the negative PSi nanoparticles in both types. PYY3-36 was released at a slower rate from THCPSi NP (80% vs. 50% from TOPSi-IMC-PYY vs. THCPSi-IMC-PYY in 8 h) due to the hydrophobicity and slow wettability of THCPSi surface, while there was no difference in IMC release from THCPSi and TOPSi (indicating its superficial independence) [91]. In addition, compared to the single-loaded model, the co-loaded model had a much greater overall release rate of both medications. Nanoparticles become more hydrophilic in the presence of PYY3-36, resulting in enhanced rates of IMC release. Enhanced and accelerated diffusion of smaller IMC molecules from the pores, in turn, caused the faster release of PYY3-36 [50].

Lim et al. synthesized a mesoporous silica-based nanoparticle suitable for the co-delivery of the hydrophobic ibuprofen (ibu) and hydrophilic acetaminophen (acet). The porous MSN loaded with the drugs and fluorescein isothiocyanate (FITC) in the pores was then coated with layers of polydopamine (PDA) and graphene oxide (GO) to give the final FMSN-drug@PDA@GO NPs with a roughened surface. Due to intermolecular interactions between the drugs and the carrier (π–π stacking, hydrogen bonding, or electrostatic retardation), the release rate profiles of the two drugs are displayed as follows: FMSNs-drug > FMSNs-drug@PDA > FMSNs-drug@PDA@GO. A release rate of 90% was reported for the FMSN-ibu and FMSN-acet after 36 h and 15 h, respectively. Nevertheless, when the nanoparticles were coated with a layer of PDA, the release rate of drugs for both individually loaded nanoparticles showed no difference, which is explained by the blockage on the outer layer of the particles, slowing the transmission of acetaminophen due to the π–π stacking interactions of the π bonds in its structure with aromatic groups in PDA. The release kinetics was then further analyzed, where the drug in FMSN-ibu@PDA@GO displayed slower release rate than the release rate of the acetaminophen from FMSN-acet@PDA@GO. The reason for this phenomenon is explained by the negative charges of ibuprofen and GO in pH 7.4, while both PDA and acetaminophen are neutral. Therefore, the transmission of the negatively charged drug through the negatively charged coating of GO may be retarded electrostatically. Moreover, regardless of pH, π–π stacking interactions with the PDA layer dominated neutral acetaminophen release, while pH alterations greatly affected negative ibuprofen release. Ibuprofen released more quicker at an acidic pH of 5.5 due to the lack of electrostatic or disrupted π–π interactions between the PDA and GO layers [92]. 

The co-delivery of genes and drugs is also an effective method of addressing various diseases. Simultaneous gene and drug delivery may increase the sensitivity of tumor cells to typical proapoptotic medicines and lower the number of chemotherapeutic agents [93,94,95]. Therefore, Babaie et al. designed 100–150 nm rod-shaped mesoporous silica nanoparticles PEGylated and functionalized with AS1411 DNA aptamer for the co-delivery of the camptothecin as a model drug and survivin shRNA expressing plasmid (iSur-DNA) in selective therapy of colorectal adenocarcinoma. Amine-functionalized MSN nanoparticles were loaded with camptothecin with 32% encapsulation efficiency due to the porous structure of the particles, in addition to the electrostatic interactions of amine groups of mesoporous silica with oxygen and nitrogen groups of CPT drug. Following the PEGylation of the drug-loaded particles using EDS/NHS technique, the particles were further tagged with the aptamers. AS1411, a 26-base guanine-rich DNA aptamer, has strong affinity and selectivity for the surface nucleolin protein, which is widely expressed in the cytoplasm and surface of many cancer cells [96,97]. iSur-DNA and the CPT drug were evaluated for their in vitro release properties. Both the 7.4 pH PBS buffer and the 5.4 pH citrate buffer demonstrated a mild burst release of camptothecin in the first 5 h, whereas after 240 h, the release rates were 16% and 65%, respectively. The proton-rich, acidic environment of citrate buffer protonated the amine and nitrogen groups in both CPT and silica, preventing the formation of hydrogen bonds, eventually resulting in higher release rates compared to the neutral environment of PBS buffer, where hydrogen bonds were strongly connecting the CPT to the silica nanoparticle. The release profile and pattern of DNA movement across the agarose gel, on the other hand, indicated that after 24 h of incubation, naked DNA was segmented, while polyplexes were intact even after incubation for 24 h in FBS-containing conditions. It demonstrates that nucleases in FBS are capable of cleaving the naked plasmid DNA, while the condensed DNA interactions with silica NP matrixes (polyplexes) were strong enough to safeguard it from degradation by nucleases [98]. 

**Figure 2:**
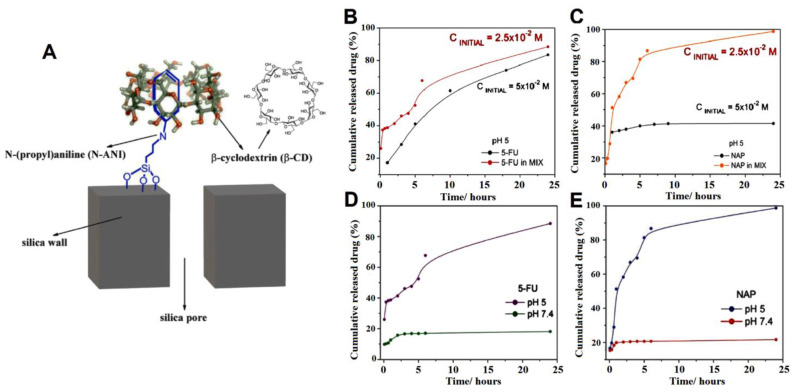
Gatekeeper interaction with silica surface and its effect on drug release in the single or co-loaded matrix. (**A**) β-cyclodextrin non-covalent interaction with N-ANI. (**B**) 5FU individual release rate comparison with mix release at pH 5. (**C**) NAP individual release rate comparison of mix release at pH 5. (**D**) 5FU release rate at pH 5 and 7.4. (**E**) NAP release rate at pH 5 and 7.4. Reproduced from Beňová et al. [100] with permission from Elsevier.

In addition to the effect of various solubilities, porous structures, diffusion rates and molecular interactions on release and loading rates and mechanisms, the capping agent effect is also an important matter to be scientifically evaluated. In a study by Benova et al., silica nanoparticles (SBA-15) were functionalized with N-[3-(trimethoxy silyl)propyl] aniline (N-ANI), then loaded with two anticancer drugs, i.e., 5-fluorouracil (5FU) and anti-inflammatory naproxen (NAP), followed by a further reaction in which the drug-loaded particle was capped with β-cyclodextrin (β-CD). β-CD is a gatekeeper used in materials that effectively caps the pores at neutral pH 7.4 but loosens in acidic environments [99]. According to the data provided by the adsorption isotherms, the loading capacity of SBA-15 nanoparticles for the co-adsorption of drugs is lower than that of SBA-15-N-ANI. Although the loading of 5FU is not significantly different between single and co-adsorption with NAP, NAP’s loading efficiency is 3-fold greater in the mixture of drugs compared to the single loading. The drug release information from the SBA-15 N-ANI-5FUN-β-CD was measured for 24 h at neutral pH = 7.4 and acidic pH = 5 via UV spectroscopy. With 1 g of nanoparticles containing 20 mg of 5FU and 65 mg of NAP, the quantity of medicine released must be calculated as a percentage relative to the total amount loaded. A total of 68% of 5FU and 87% of NAP were released in the first six hours when the pH was set to 5, indicating that the caps were presumably open. After 18 h, the accumulated release of 5FU reached 88.5%, whereas NAP’s considerably slower and more consistent release reached 98.7%. However, at pH 7.4, the drugs were released at a substantially slower rate (16% of 5FU and 20% of NAP in 3 h, and no further release). In neutral pH, the initial release is likely low because not all of the nanoparticles’ pores have been capped. Drug release profiles show that after 24 h, 84% of 5FU was released as a single drug, and 89% was released when combined with NAP. Accordingly, the release of NAP demonstrated a 40% burst release in the first hour and no further release as a single drug in acidic pH of 5, while 98.7% of NAP was released in the mixture of drugs after 24 h. The release results displayed the effective use of gatekeepers to control the release of a dual-drug system [100] (Figure 2).

**Figure 3:**
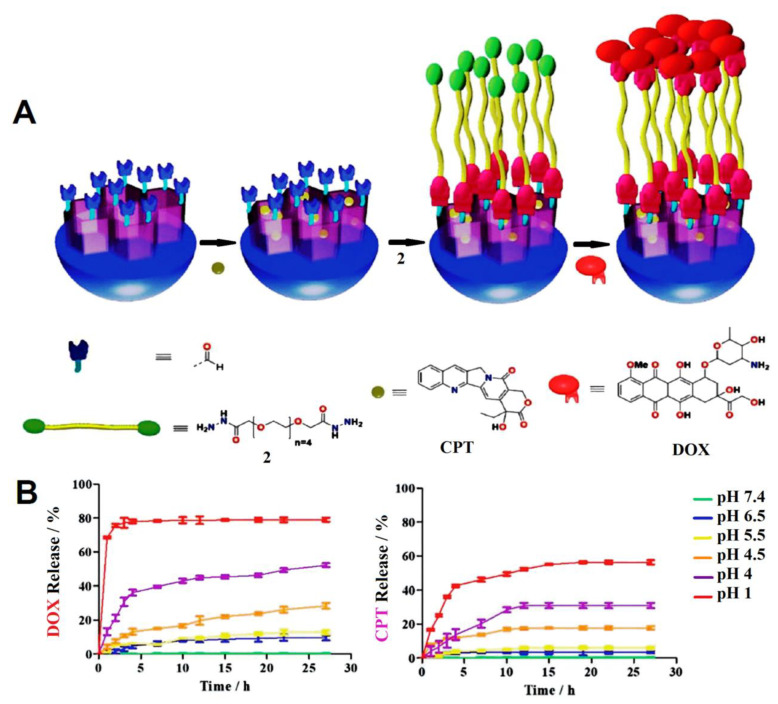
(**A**) Schematic process of capping the CPT-loaded nanoparticles with DOX. (**B**) DOX and CPT release profiles in various pH values in CPT@MSN-hyd-PEG-hyd-DOX. Reproduced from Llinàs et al. [103], © 2018, the Author(s). Published by Informa UK Limited, trading as Taylor & Francis Group.

Controlled release in dual-drug systems may also be possible using a drug as a gatekeeper. A novel system based of mesoporous silica nanoparticles was designed to deliver two drugs, DOX and CPT. The synthesis of delivery systems was carried out in three steps, where MSN-(NH_2_) CTAB was first created. After removing CTAB as a surfactant, an aldehyde group was introduced into the system to give MSN-(NH_2_)-(CHO), followed by the third step where CPT was loaded inside the pores at 3.1% efficiency in a mixture of CHCl_3_/MeOH. Then, DOX and dihydrizide PEG were used as capping agents, which also increased the hydrophilicity of the outer layer of the nanoparticles. Tetraethylene glycol chains have been previously studied as a blocking agent of the MSN pores [101,102]. The loading of DOX as the second drug and hydrophilic gatekeeper has also altered the ζ-potential of the nanoparticles to the positive charge +20.1 mV. The pH values of 1.0, 4.0, 4.5, 5.5, 6.5, and 7.4 were used in a release analysis of the final CPT@MSN-hyd-PEG-hyd-DOX. DOX showed a fast release in 20 h at 80% in pH 1.0, 45% in pH 4.0, 25% in pH 4.5, and 10% in pH 5.5 due to the breakage of the hydrazone bond in the acidic environment, although with a decreased release rate after 5 h, whereas CPT exhibited a prolonged release after 10 h due to the physical adsorption of the drug into the nanoparticles’ pores (Figure 3). Importantly, at a pH of 7.4, release for both drugs was marginal, demonstrating the capping agent’s efficiency in preventing the fast diffusion of drugs into undesired bodily sites [103].

Targeting tumor cells is a method to decrease the typical cancer therapy side effect. Therefore, a disulfide containing redox-responsive polymeric nanoparticle was synthesized. Poly alendronate-hyaluronan-S-S-curcumin copolymer (ALN-oHA-S-S-CUR) consisted of a hydrophobic core containing CUR and a hydrophilic shell containing ALN drug, and oHA-targeting CD44 receptor. For the treatment of bone disorders, alendronate (ALN) is the most prominent anti-resorber, which has an excellent binding attraction for bone minerals and limits osteoclast activity, thereby protecting bone tissue from destruction [104]. Hydrophilicity, biocompatibility, plasma stability, and CD44 receptor targeting are among some of the remarkable characteristics of oHA, a small molecule obtained from HA breakdown [105,106,107]. The disulfide bond supports the structure of the co-delivery systems by forming a micelle in water, which, upon penetrating the target cells, increased environment cleavage at the S-S bond, releases the drugs. To determine the function of the micelles in the presence of reducing agent, the drug-containing particles were studied in varying concentrations of L-glutathione (GSH), where nearly 70% cumulative drug release was obtained in 10 mM GSH compared to 30% release in 1 mM GSH after a 70 h duration. Furthermore, the stability of ALN-oHA-*S*-*S*-CUR particles was examined during the release of CUR and in the presence of 30 mM dithiothreitol (DTT) in PBS. The results showed that in the solution containing DTT, the CUR precipitated more than the control solution without DTT, which indicated the sensitivity of the CUR-loaded particles to the reduction environment [108].

Cleavage of disulfide bonds in the polymeric structure as the means of dual-drug release and diffusion was also conducted in other studies. Lipid–polymer hybrid nanoparticles (LPNs), with polymeric cores and lipid/lipid–PEG shells, integrate the physicochemical characteristics and biocompatibility of polymeric nanoparticles and liposomes [109,110,111]. Increased biocompatibility and slowed drug release are all benefits of the lipid shell and by incorporating a second lipid–PEG layer into the lipid shell, hydrophilicity at the surface, protection from RES, steric stability, and enhanced blood circulation might all be achieved [112]. In this regard, the lipid–polymer nanoparticle using poly (ethylene glycol)-*S*-*S*-hexadecyl (mPEG-*S-S-*C_16_), soybean lecithin, and poly (D, L-lactide-co-glycolide) (PLGA) was designed to convey and release doxorubicin (DOX) and triptolide (TLP) drugs by sensitivity to reduction. Triptolide (TPL/TL), also known as tripterygium wilfordii lactone alcohol or tripterygium wilfordii lactone, is a compound derived from the tripterygium wilfordii celastraceae plant. Multiple cancer cell lines have shown in preclinical research that TPL promotes cell death [113,114,115], decreases tumor spread and suppresses cell proliferation [116,117], and boosts the efficacy of other treatment techniques [118]. Accordingly, the systems’ structure comprises of the PLGA hydrophobic core, the lecithin monolayer, and mPEG-*S-S-*C_16_ as the outer layer to evaluate the effect of dual delivery of the drugs on human oral cavity squamous cell carcinoma cells (KB cells). For creating the suitable structure, including an intracellular release under reductive conditions, the reduction-sensitive polymer mPEG-*S*-*S*-C_16_ was integrated into PLGA-lecithin, where PEG fragments were eliminated from LPNPs containing mPEG-*S*-*S*-C_16_, leaving the remaining nanoparticles unstable and resulting in rapid release of drug to the hydrophobic portion.

**Figure 4:**
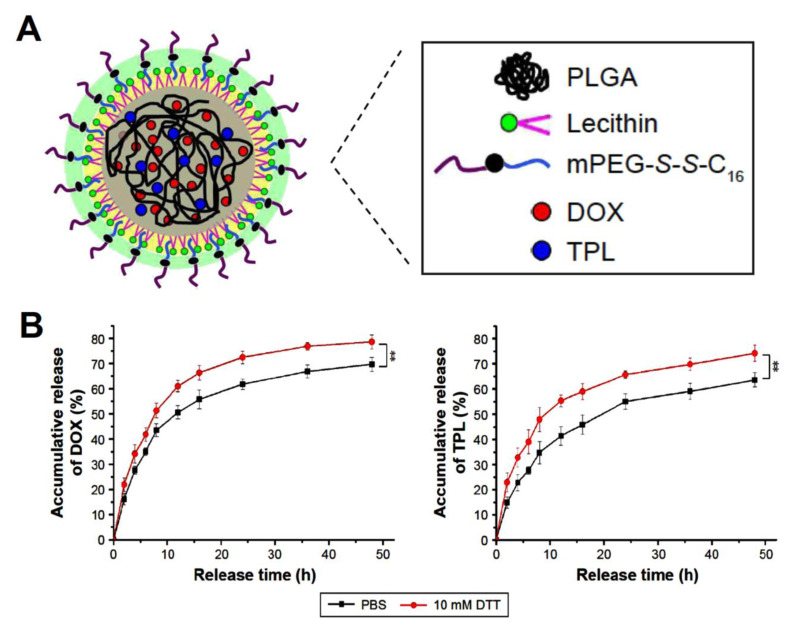
Lipid–polymer hybrid nanoparticles containing S-S bond sensitive to a reductive agents/environment. (**A**) LPNPs core–shell design including a hydrophobic core for loading of both TPL and DOX drugs. (**B**) Release profiles of DOX and TPL from DOX/TPL-1/0.2-loaded LPNPs in PBS at pH 7.4 in the presence of 10 mM DTT and in the absence of DTT (** *p* < 0.05). Reproduced from Wu et al. [119], International Journal of Nanomedicine 2017 12 1853-1862. Originally published by and used with permission from Dove Medical Press Ltd.

The spherical core–shell structure indicates a hydrophobic sphere core suitable for the entrapment of drugs and compounds with poor water solubility. DOX and TPL, at a weight ratio of 1:0.2, exhibit an encapsulation efficiency of 75.5% and 58.3%, respectively. Moreover, the drug release profiles were studied both without and with DTT as reducing agent. Increased drug release was seen in DTT-containing environments compared to PBS, with DOX release reaching 78% in the DTT-containing solution and 64% in the PBS-only control. TPL was also released in similar patterns after 48 h in both the PBS buffer and the DTT solution (62% vs. 70%, respectively) (Figure 4) [119].

For taking advantage of the protein-based and lipid-based nanoparticles properties in drug delivery systems, Amer Ridha et al. reported the synthesis of a novel protein–lipid complex nanoparticles with protein outer layer and lipid core. This drug delivery system was used for simultaneous delivery of doxorubicin (DOX) and mitoxantrone (MTO); which the latter is an anthracycline antibiotic with a broad-spectrum anticancer activity that may intercalate DNA and disrupt topoisomerase II [120]. The system was built in two individual stages: First, DOX was loaded in the interior space of the Apoferritin (AFr) protein with the help of its capability to form a hollow cage upon self-assembly, which dissociates into its constituent parts at a pH of 2.0 and reforms at a pH of 7.4 [121,122], followed by functionalization with folic acid (FA) receptor to give the final TPN particle with a negatively charged surface. Second, the MTO was encapsulated with 97% EE in the cationic solid lipid nanoparticles (cSLN) as a class of SLNs with positive surface charge and the ability to convey various cargos [123,124]. The ultimate dual-targeted protein–lipid nanocomplexes (DTPLNs) were constructed by assembling two complexes through ionic interactions. The in vitro drug release data were also evaluated under different conditions. At an acidic pH of 6.8 and in the early stages, MTO released faster than pH 7.4 from cSLN, which may be due to the instability of lipids in acidic environments compared to neutral one. A similar release pattern was observed for the release of DOX from TPN at an acidic pH of 6.8, where almost all DOX was released from the nanoparticles as a result of deteriorated hydrogen bond between hydroxyl groups in DOX and amine groups of AFrs. Regarding the electrostatic attachment of TPNs to MTO-cSLNs, the protein ionic charge in DTPLNs was at its lowest at pH 6.8, diminishing the electrostatic attraction of protein to cSLNs and speeding up MTO release. Over 72 h, DTPLNs released 75.99% of encapsulated MTO at pH 6.8 and 68.92% at pH 7.8, whereas DOX released 94.52% at pH 6.8 and 59.52% at pH 7.4. The findings demonstrate that the co-loaded mechanism could release a higher amount of drugs in the lower pH environment typical of the region around a tumor [125]. 

### 2.3. Internalization and Cytotoxicity Data 

Passive or active targeting can both lead to nanocarrier aggregation in cancer cells. Bio-functionalizing the surface of NPs with ligands, proteins, aptamers, and polysaccharides that bind with high affinity to overexpressed receptors on tumor cells or secreted proteins in the tumor microenvironment (TME) is an example of active targeting [126,127,128]. For instance, due to the high affinity of the folate ligand for the folate receptor, which is overexpressed in cancerous cells, the use of folate-conjugated nanoparticles in drug delivery systems has received much attention [129,130]. Using a dual system of FA-DOX/siRNA-L liposomes, the co-delivery of DOX (a chemotherapeutic drug) and Bmi1 siRNA (a siRNA having Bmi 1 gene silencing capabilities) has been investigated by Yang et al. DOX-L (DOTAP/Chol/mPEG-DSPE) and FA-DOX-L (DOTAP/Chol/mPEG-DSPE/FA-PEG-Chol) encapsulated Bim1 via electrostatic interactions. (mPEG-DSPE: monomethoxy polyethylene glycol 2000-distearoyl phosphatidylethanolamine; Chol: cholesterol; DOTAP: 2-dioleoyl-3-trimethylammonium-propane (chloride salt)). The internalization of folate-conjugated liposomes carrying only siRNA or DOX was much greater than that of liposomes devoid of folate ligand, as shown by fluorescence examination of three cell lines (KB, HeLa, and Hep3B). More so, the uptake of the medicines in cells was much higher in FA-DOX/siRNA-L particles, showing the increased targetability of the liposomes owning folate ligand, compared to DOX/siRNA-L particles. FA-DOX/siRNA-L was also validated for its improved cytotoxic qualities, where 90.5% of KB cells, 82% of HeLa, and 68% of Hep3B were killed, much greater than the rates attained by FA-siRNA-L or FA-DOX-L alone. The KB xenograft tumor mouse model was also used to examine the suppression of tumor proliferation. Among all variants of liposomal formulations, the FA-DOX/siRNA-L groups had a significant reduction in tumor size after 25 days of injection, along with no toxicity and weight loss results, unlike other tested formulations. Mice treated with FA-DOX/siRNA-L showed greater apoptotic capacities, as evidenced by stronger signals in TUNEL assays compared to mice treated with FA-DOX-L or FA-siRNA-L solely. Based on the known functions of p14 and p21 in triggering cellular death and p16 in triggering growth arrest, the q-RP-CRT results implied that Bmi1 knockdown partially participates in the apoptosis and growth suppression of cancer cells [131]. 

The use of folate ligand for selectivity to cell receptors was also observed in a more recent study where the magnetic nanoparticles possessing folate and fluorophore fluorescein were further functionalized with β-CD-MA-PNIPAM copolymer through urea bond formation, loaded with the widely used combination of drugs, curcumin and doxorubicin. Notably, hydrophobic curcumin was in the polymeric matrix and cyclodextrin cavity. Despite being hydrophilic, DOX is confined in the polymeric matrix rather than the cyclodextrin cavity. Various test groups were evaluated for MTT assay utilizing the Hela cell line. Accordingly, the cell viability was the lowest for drug-loaded nanoconjugates among all groups, while nanoconjugates showed lower toxicity. Cell viability was even lower with the use of a magnet, indicating the desired performance of drug-loaded nanoparticle, and explained by the fact that the nanoconjugates are superior in their ability to induce endocytosis and hence deliver large amounts of drugs into the cell. After being taken up by cells, curcumin accumulates in the ER membrane, depletes the cell’s iron supply, and potentially causes cell death. The fluorescence analysis data also displayed that internalization was greatly improved by conjugation with folate compared to nanoconjugates devoid of folic acid. Additionally, the results showed that the internalization of nanoconjugates increased somewhat in the presence of a magnet. Propidium iodide (PI) labeling, known for the capacity to only penetrate dead cells, was utilized to detect cell death rate, where the presence of PI-positive cells upon injection of curcumin and DOX evidenced the significant cell death in this investigation. The most notable effect was observed with nanoconjugates containing drugs and an increased number of PI-positive cells. Furthermore, in vivo testing on a tumor-induced mouse model of HCC (Hepatocellular carcinoma) confirmed the successful functioning of the folate-functionalized nanoconjugate. For instance, in models treated with DOX-loaded nanoconjugates, there was no change, a decrease, and a lower trend in body weight (as an indication of the tumor growth in the liver), SGPT and SGOT liver serums, and Alpha fetoprotein (AF). However, the patterns for these parameters were raised and higher in just DOX-treated models. Furthermore, DOX-loaded nanoparticles significantly reduced inflammatory indicator levels (TNF-, MCP-1, IFN-, IL-6, IL-10, and IL-12), MMP-2, and 9, compared to only DOX groups, highlighting that the beneficial impacts were attributed to the targeted delivery of drugs by nanoconjugate [132]. 

**Figure 5:**
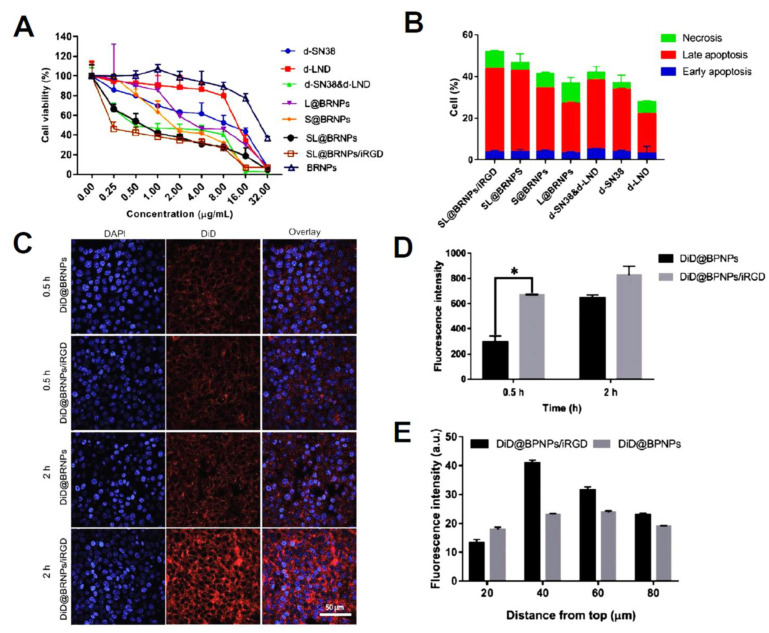
(**A**) Cell viability by MTT assay. (**B**) Apoptosis results from various groups. (**C**) 4T1 cells treated with nanoparticles for 0.5 and 2 h by confocal microscopy. (**D**) Flow cytometry data of cellular uptake. (* *p* < 0.05) (**E**) Analysis of tumors at various depths via fluorescence semi-quantification. Reproduced from Yang et al. [136], with permission from John Wiley and Sons.

Due to its chemical structure, PEG renders NPs more stable in biofluids, lessens their aggregation, and limits NPs’ connection with not-specific proteins, leading to a “stealth” state that can enhance the circulation time of PEG-NPs while minimizing phagocytosis [17,133,134]. Using cell-penetrating peptides is another method that has received much attention for its potential to boost NPs uptake. These molecules, often containing polycationic or amphipathic structures, are constructed of a particular aminoacidic sequence that facilitates the absorption of NPs [135]. For instance, a 2019 study reported a self-assembled PEGylated bilirubin which encapsulated disulfide-linked dimeric prodrugs of dimer-7-ethyl-10-hydroxycamptothecin (d-SN38) and dimer-lonidamine (d-LND) [136]. Apparently, LND is a hexokinase inhibitor that demonstrates therapeutic effectiveness by affecting metabolic activity [137], whereas SN38, an active component of camptothecin in vivo, operates on DNA topoisomerase, enabling carcinoma cell death [138,139]. In addition to PEG, the nanoparticles also featured the tumor-penetrating peptide iRGD (cRGDKGPDC), which binds exclusively to integrin receptors α_v_β_3_ and α_v_β_5_. The usage of anti-PD-L1 antibody in the systems also had the combination of chemo and immunotherapy against breast cancer cells. The effectiveness of the iRGD-decorated nanoparticles was determined using fluorescence imaging, and the findings showed that the iRGD-containing group’s uptake in the 4T1 cell line resulted in higher fluorescence intensities than the control group. In order to evaluate the formulations’ capacity to penetrate tumors, tumor spheroids were used, and semiquantitative fluorescence intensities revealed that the iRGD group had the most enrichment occurring at a depth of 40 µm, demonstrating increased penetration. In addition, more accumulated iRGD group at tumor sits was observed compared to control group, indicating successful targetability (Figure 5). MTT assays of SL@BRNPs/iRGD with IC_30_ of 0.47 µg mL^−1^ compared to single-drug-loaded and free dimer or normal drugs indicated the lowest cell viability and presented higher toxicity. 

Similarly, 54.2% of cells apoptosis was observed in SL@BRNPs/iRGD groups, which was 1.12-fold greater than SL@BRNPs, demonstrating the superior absorption of nanoparticles by iRGD and the subsequent increase in cell death. The anti-tumor efficiency of nanoparticles was higher when used with anti-PD-L1 as a tumor growth inhibitor, and improved tumor suppression better than groups with a single drug or without iRGD. CD8+ T cell activation by anti-PD-L1 was further observed by improved data from SL@BRNPs/iRGD + anti-PD-L1 group among other control groups, using flow cytometry [136]. 

**Figure 6:**
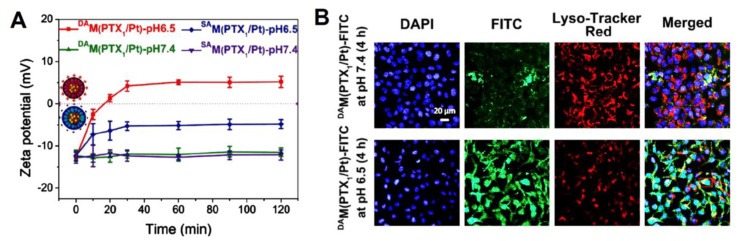
(**A**) Zeta potential conversion of DA and SA-containing micelles through pH variations. (**B**) CLSM images Skov-3 incubated with FITC-conjugated micelles in pH 7.4 and 6.5. Reproduced from Liao et al. [140], with permission from the Royal Society of Chemistry, Great Britain.

In another study, poly (AMA-co-IMMA)-b-poly(OEGMA) (PAIPO) copolymers conjugated with paclitaxel (PTX) and cis-platinum (Pt(II)) drugs, owned PEG in the polymer chain, to enhance circulation time in bodily. However, this dual delivery system used a novel method for internalization, cytotoxicity, and biocompatibility improvement, namely the change in surface charge owing to the sponge effect of imidazole ring in the micelle structure though deprotonation in neutral pH and protonation in acidic pH of 6, close to that of the endocytic environment, causing osmotic swelling and membrane disruption, eventually contributing to release of drugs. Moreover, micelles’ surface ionic groups can alter from −COO− to −NH_3_^+^ due to conjugated 2,3-dimethylmaleic anhydride (DA) group under pH below 6.5. Consequently, the zeta potential of micelles might switch from negative to positive, given the pH difference surrounding the tumor site. It is also worth to mention the existence of GSH-sensitive S-S bond and ascorbic acid-sensitive amino bond, conjugating PTX and Pt (II) to the polymer chain, improving the precise tumor site release of the drug. Despite ^DA^M(PTX_1_/Pt) (DA-containing micelle) and ^SA^M (PTX_1_/Pt) (succinic anhydride (SA)-containing micelle) negative charges at pH 7.4 and 6.5, the zeta potential of ^SA^M(PTX_1_/Pt) slowly increased with incubation at pH 6.5 in the first 30 min up to −5.0 mV after 120 min. However, the zeta potential of ^DA^M (PTX_1_/Pt) rapidly increased from −12.80 ± 1.27 to +4.24 ± 1.22 mV with incubation at pH 6.5 in the first 30 min and reached +5.23 ± 1.35 mV after 120 min, suggesting the successful conversion of –COO− groups to –NH_3_ in the DA molecules upon pH variation (Figure 6). Cell-viability data of the HeLa and Skov-3 cell lines demonstrated the highest synergic effect in ^DA^M(PTX_n_/Pt), exhibiting the lowest IC_50_ = 0.37–0.79 for HeLa, and 0.41–0.78 for Skov-3. Confocal microscopy images indicated that after 4 h of incubation at pH 7.4, only a few amounts of ^DA^M (PTX_1_/Pt)-FITC could be seen in the cytoplasm, indicating that only a few cancer cells had taken up the dye. The charge-conversion performance supplied by DA moieties clearly revealed that they might increase the cellular uptake of ^DA^M (PTX_1_/Pt)-FITC at pH 6.5, as seen by the significantly greater green fluorescence observed during internalization (Figure 6). According to the CCK-8 assay, the ^DA^ M (PTX /Pt) resulted in nearly greater apoptotic rates of 18.8% and 34.5% in Skov-3 and HeLa cells, respectively, compared to other groups of free single drugs and combination groups. All data confirm the suitable dual delivery of the anti-tumor drugs using the micelles decorated with charge-converting agents [140].

Including poly(2-ethyl-2-oxazoline) (PEOz) in the structure of carriers could prolonged the circulation time and enhanced cell uptake through charge conversion from negative to positive under lysosomal and endosomal. Black phosphorus (BP) sheets with a high surface area and optoelectronic properties were used as the basic component and loaded with DOX as the first anticancer medication. The nanosheet was subsequently coated with polydopamine (PDA) to prevent the BP from degrading in aqueous media. The resultant particles were subsequently functionalized with PEOz instead of the conventional PEG and loaded with bortezomib (BTZ) to form chemo-photothermal nanoparticles (BP-DOX@PDA-PEOz-BTZ). Changing the pH from physiological pH (7.4) to more acidic pH (6.8 and 5), the surface charge in BP-DOX@PDA-PEOz-BTZ converted from negative to positive while the nanosheets with PEG remained negative. Confocal laser scanning microscopy was used to demonstrate MCF-7 cells taking up BP-DOX@PDA-PEG and BP-DOX@PDAPEOz nanosheets at pH 7.4 and 6.8. The intracellular fluorescent intensities of BP-DOX@PDA-PEG NSs at pH 6.8 and 7.4 were comparable after 4 h of treatment, while the intensity of BP-DOX@PDA-PEOz nanosheets at pH 6.8 greatly surpassed that at pH 7.4, confirming that PEOz enhanced the cellular absorption of DOX, possibly due to the charge reversal of PEOz when tertiary amide groups throughout the PEOz chain were ionized. Cytotoxicity in vitro and photothermal behavior of drug-free BP@PDA-PEG and BP@PDA-PEOz nanosheets, as well as DOX-loaded BP@PDA-PEG, BP@PDA-PEOz, and BP@PDA-PEOz-BTZ nanosheets, were evaluated using the MTT assay. Although NIR laser irradiation alone had a negligible effect on cell proliferation, BP@PDA-PEG, and BP@PDA-PEOz nanosheets had photothermal effects that varied with concentration. When 50 mg/mL of BP@PDA-PEOz was used along with 808 nm laser light, over 80% of MCF-7 cells were killed, demonstrating the potential usage of BP@PDA-PEOz as a photothermal agent. Further, BP-DOX@PDA-PEG exhibited comparable inhibitory effects at pH 7.4 and 6.8, whereas BP-DOX@PDA-PEOz was much more cytotoxic at pH 6.8. PEOz modification promoted tumor inhibition by enhancing cellular absorption and pH-sensitive drug release in moderately acidic tumor tissues. MCF-7 cells were treated with DOX, BTZ, DOX + BTZ (1:1), and drug-loaded nanosheets at various DOX dosages. The cytotoxicities of free DOX, BTZ, DTX + BTZ (1:1), and drug-loaded nanosheets were time- and dose-dependent, with co-administration of DTX + BTZ being more cytotoxic than DOX or BTZ alone. After 24 or 48 h of incubation, cells treated with DOX-loaded BP@PDA-PEOz nanosheets exhibited a lower survival rate than those treated with PEG nanosheets, indicating that PEOz extended drug circulation in vivo more than PEG, increasing drug half-lives at tumor sites. Most notably, DOX-loaded BP@PDAPEOz-BTZ with 808 nm laser irradiation (1.0 W cm^−2^) had the lowest rate of survival after 48 h, indicating that therapy with photothermal therapy was the most toxic [141].

In order to combat the high mortality rate associated with glioblastoma multiforme (GBM), a particularly aggressive form of brain tumor, gold nanoparticles loaded with 5-fluorouracil were encapsulated with chitosan (CS). The NPs were then functionalized with the AS1411 aptamer through a phosphoramidated bond, inducing targetability towards nucleolin proteins overexpressed on cancerous cells [142], and thereafter DOX was encapsulated. Flow cytometry data of the 5′6-FAM tagged Apt-DOX-CS-Au-5FU NPs incubated with LN229 cells demonstrated 98.17% fluorescent intensity, much higher than the DOX-CS-Au-5FU NPs and free DOX, displaying the importance of aptamer functionalization in achieving enhanced uptake. These data were further approved using HR-TEM, where the more internalized AuNPs were attributed to the aptamer-functionalized nanoparticles. The Apt-DOX-CS-Au-5FU NPs also demonstrated the highest cytotoxicity against LN229 cells in addition to high proliferation inhibition induced by dual-loaded NPs. More necrotic cells were observed in the DOX treatment, while Apt-DOX-CS-Au-5FU NPs promoted apoptotic cell death, as determined by the IC50 concentrations of the different nanoparticles and drugs used to treat LN229 cells. Flow cytometry showed that DOX and 5FU increased necrotic cells by 63.6% and 56.19%, respectively, demonstrating that necrosis was the major cell death mechanism of the free anticancer medicines. In total, 12.69% apoptosis and 3.11% necrosis were found in cells following treatment with AuNPs alone. Interestingly, cells treated with 5FU-loaded AuNPs demonstrated 10.84% apoptosis and 18.66% necrosis and CS-coated Au-5FU NPs lowered necrosis by 3.49% and apoptosis by 18.3%. Apt-functionalized DOX-CS-Au-5FU NPs also exhibited less necrosis (7.03%) and more apoptotic cells (23.11%) than the drug alone and other nanoparticles. Accordingly, the necrosis mechanism is reported to be responsible for the death of the cells through inflammation rather than the suppression of cancerous cell growth [143].An additional indicator of the proliferation inhibition induced by Apt-functionalized DOX-CS-Au-5FU NPs in the apoptosis mechanism was confirmed via less necrosis (7.03%) and a higher percentage of apoptotic cells (23.11%) compared to free drug and other groups [144].

Through a layer-by-layer approach, the same drug combination as the previously mentioned study was encapsulated in a nanoparticle of chitosan/dextran/chitosan (CS/DEX/CS), with the first layer consisting of chitosan and paclitaxel (PTX) formed via the double-emulsion method. Additionally, dextran was electrostatically deposited onto the nanoparticles, creating a negatively charged coating. Chitosan was used once more for the final layer, this time encapsulating the 5FU medication to create CS-PTX/DEX/CS-5Fu NPs. Zeta-positive chitosan with amine groups that enables rapid uptake into cells via endocytosis may be complexed with negatively charged dextran sulfate (DEX) through polyelectrolyte complexation and form multilayers nanoparticles via self-assembly, suitable for cargo delivery purposes. In the process of nanoparticle synthesis, the zeta potentials of each layer altered from 33.7 ± 5.4 mV for CS-PTX to −24 ± 13.9 mV for CS-PTX/DEX, and 24.8 ± 9.4 mV for CS-PTX/DEX/CS-5FU, illustrating successful coating of each layer. MTT evaluation of dual drug-loaded nanoparticles plus PTX-loaded nanoparticles against HepG2 cells revealed the greatest cell inhibitory activity for CS-PTX/DEX/CS-5Fu in comparison with single-drug groups, due to the enhanced absorption of the dual drug-loaded nanoparticles, leading to a synergistic lethal effect. Importantly, electrostatic adsorption contributed to the uptake of positively charged CSPTX/DX/CS-5Fu NPs into negatively charged cancer cells. Moreover, the inhibition ratio for CS-PTX/DEX/CS NPs without 5FU was 43% after 36 h and 44% after 72 h of in vitro cellular uptake time, while the cytotoxicity reported for multilayered nanoparticles loaded with both drugs increased to 56% after 36 h at a concentration of 5 g/mL and 55% after 4 h at a concentration of 30 g/mL. Additionally, according to apoptosis assays using tracking of FITC containing groups of PTX, CS-PTX, CS-PTX/DEX, CS-PTX/DEX/CS-5FU, and empty multilayered nanoparticles evidenced a higher apoptosis rate of 39.55% for the dual-drug-loaded nanoparticles, apparently more than all drug-containing groups, while no to negligible apoptotic activity was observed for control and empty nanoparticles [145]. 

In a different study, 5-fluorouracil (5FU) was also used to treat colorectal carcinoma (CRC), one of the deadliest cancers. However, in the interest of controlling the multidrug resistance (MDR) caused by long-term therapy with 5FU, this medicine was administered in combination with miR-21 (miRNA), which has demonstrated effective anti-drug resistance properties [146]. Exosomes with qualities such as penetrating biological barriers (e.g., the blood–brain barrier), transport of functional cargos into cells, and blood stability [147] were employed as the nanocarriers for the co-delivery of the miR-21 inhibitor and 5FU into 5FU-resistant HCT-116(HCT-1165^FR^) cells, where miR-21 is overexpressed. Her2-binding affibody was fused to the Lamp2, a human protein in exosomal membranes, and then transferred into pLVX-GFP-N1. The resulting fusion protein (THLG) consists of Her2-binding affibody, LAMP2, and GFP. The Her2-LAMP2 fusion protein was placed on the surface of the exosomes containing the cargos, allowing for targeted cellular uptake via EGFR receptor-mediated endocytosis in HCT-116 cells. A co-culture model consisting of Her2-negative SGC-7901 WT cells and Her2-positive Her2-mcherry-SGC-7901 cells was used to investigate the in vitro targeting potential of THLG-exosomes (THLG-EXO). Following three hours of co-culture with THLG-EXO, fluorescence microscopy indicated that THLG-EXO penetrated Her2-mcherry-SGC-7901 cells more efficiently than SGC-7901 WT cells. Using T-Her2 as the ligand for Her2 significantly enhanced the binding ability of exosomes to target cells, as demonstrated by the fact that THLG-EXO with targeting Her2 proteins on their surface facilitated the uptake of THLG-EXO by cells. A significant reduction in tumor volume was seen in the group treated with the THLG-EXO/5FU/miR-21i formulation in nude mice with HCT-1165FR-Luc (luciferase expressing) tumors. Compared to the THLG-EXO and THLG-EXO/5FU groups, the THLG-EXO/5FU/miR-21i group’s tumor development was greatly suppressed, and the tumor weights of their mice were much lower. The results of TdT dUTP nick end labeling (TUNEL) staining in comparing the levels of apoptosis indicated that injections of THLG-EXO/miR-21i caused a modest increase in TUNEL-positive cells, indicating that miR-21i alone had a minimal effect on cell death while exosomes containing miR-21i in combination with 5FU might generate a potent anti-tumor impact. Moreover, THLG-EXO/miR-21i and THLG-EXO/5FU/miR-21i rescued the protein expression of hMSH2 and PTEN in the tumor tissue, but the singly administered 5FU via THLG-EXO had almost no detectable effect on the expression of hMSH2 and PTEN. Overall, the results showed a potent synergism owing to the THLG-EXO-mediated co-delivery of miR-21i and 5FU [148].

**Figure 7:**
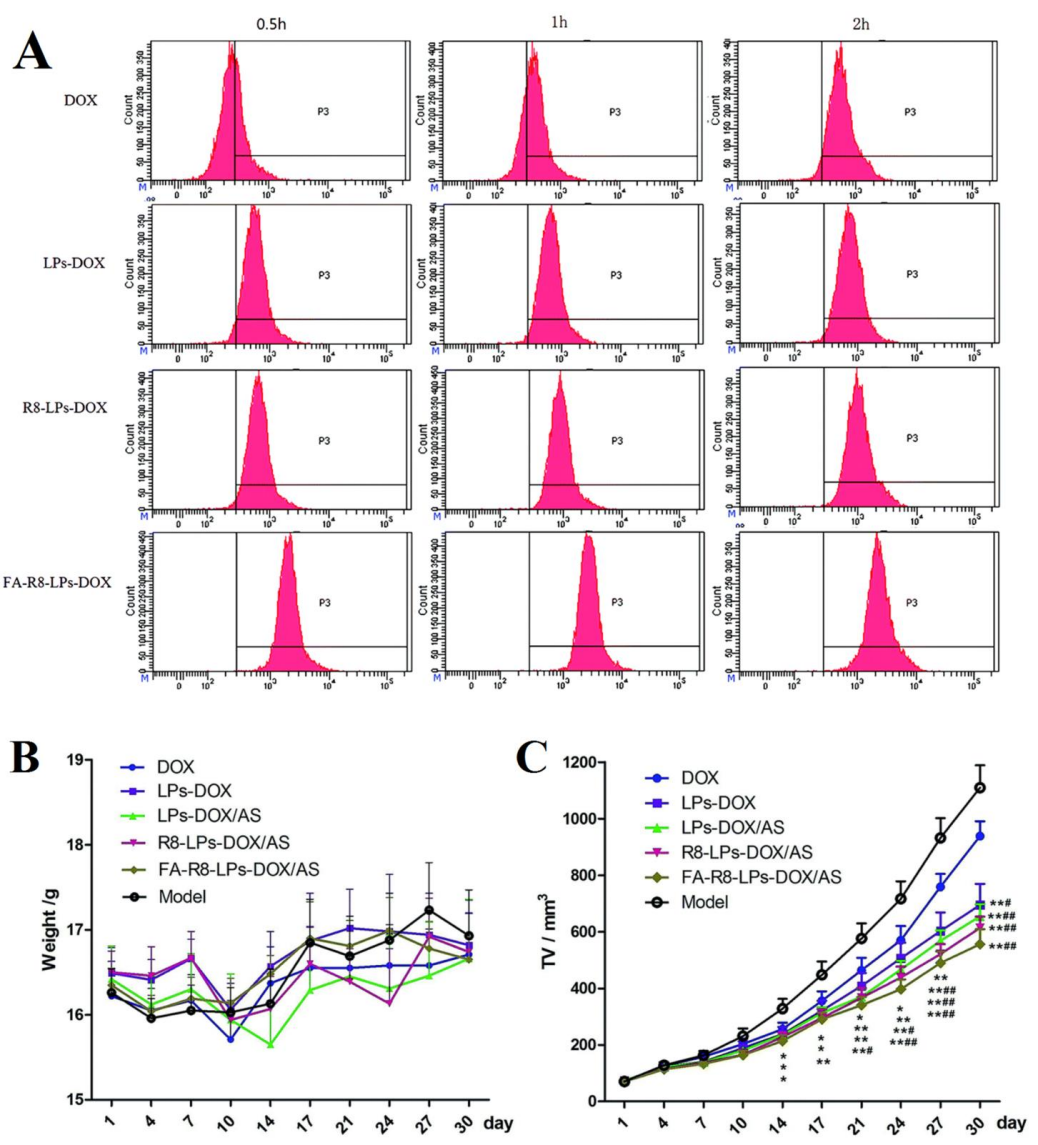
(**A**) Flow cytometry diagrams in different time intervals. (**B**) Changes in body weight in mice treated in different test groups. (**C**) Changes in tumor volume treated in different test groups (in comparison to control group with * *p* < 0.05 and ** *p* < 0.01 and DOX group with # *p* < 0.05 and ## *p* < 0.01). Reproduced from Yue et al. [150]. © The Royal Society of Chemistry 2020.

For the purpose of treating triple-negative breast cancer, liposomes functionalized with targeting agents were used to co-load doxorubicin (DOX) and astragaloside IV (AS-IV). AS-IV, acting as a resistance reversal moiety, increased the inhibitory action of DOX, while folate ligands for folate receptors were decorated on the surface of the liposomes through octa-arginine polypeptide (R8), which not only acted as a linker but also penetrated any cell membrane [149], improving the internalization and targetability of the final FA-R8-LPs-DOX/AS formulation. The flow cytometry results of the in vitro internalization suggested the higher uptake of DOX from the double functionalized FA-R8 liposomes in the MDA-MB-231/DOX than free DOX or DOX-LP. This was also confirmed by confocal laser scanning microscopy images where the fluorescent intensity of DOX around the nucleus was greater for DOX from FA-R8-LPs, indicating the optimal function of FA and R8 in penetration and accumulation of DOX in the vicinity of the nucleus (Figure 7). Further, the in vivo targetability of the dye-conjugated groups was evaluated where the intensity of the groups was highest for FA-R8-LP/DIX. Using the MTT test, we saw that the DOX resistance of MDA-MB-231/DOX cell lines decreased, from an IC50 of approximately 5M to 2M following the insertion of AS-IV, hence increasing the efficiency with which DOX inhibited cell growth. Nude mice treated with FA-R8-LPs-DOX/AS lost very little weight, demonstrating the specific impact of anti-tumor medications and their safety for healthy tissues (Figure 7). Tumor volume also was smallest after two weeks of treatment with FA-R8-LPs-DOX/AS, followed by R8-LPs-DOX/AS and LPs-DOX/AS. The co-loaded, double-functionalized liposomes clearly showed the highest anti-tumor activity, making them suitable for use against breast cancer [150].

All mentioned research data of co-delivery systems via uniform structures are summarized in Table 1.

## 3. Anisotropic Structures

Changes in the surface structure of nanoparticles enable simultaneous drug administration with varying efficiencies, depending on the intended purpose of drug delivery and the required particular properties in solubility, interactions, and drug release. Heterogeneous engineered structures include Janus particles with two distinct portions (two-faced NPs), patchy nanoparticles with structural heterogeneity in the shell, and multicompartment nanoparticles with heterogeneity mostly focused in the nanoparticles’ cores. The heterogeneous components of these structures let diverse features, such as surface charge, morphology, functional groups, and hydrophobicity or hydrophilicity, exist in a single structure, therefore facilitating the storage of pharmaceuticals in different regions of the heterogeneous structures. The following section exemplifies the key aspects of two-drug delivery utilizing anisotropic structures, contributing to a better understanding of the co-delivery systems and the advantage of using anisotropic particles.

### 3.1. Solubility and Encapsulation

Given their two-sided structure, a wide variety of drug delivery issues, such as the simultaneous encapsulation of medicines with dramatically varying solubilities, may be susceptible to solutions with the help of Janus particles. In contrast to single-agent chemotherapies, this category of nanoparticle has the advantage of co-delivering numerous therapeutic chemicals with controlled release rates to targets for synergistic therapeutic effects [151]. 

Using the Pickering emulsion method, Xing et al. synthesized the inorganic–metallic structure of Janus particles. Wax droplets contained mesoporous silica spheres, which enabled the formation of a gold hemisphere on the side outside the wax. In the next step, the hydrophilic drug DOX was encapsulated on the silica side of the half-coated particles, while the hydrophobic drug PTX was attached to the Au half by functionalizing it with thiol-β-cyclodextrin (SH-β-CD) [152], which helped with enhanced PTX solubility due to the complex formation between PTX and the hydrophobic thiol-β-cyclodextrin domain [153].

Chen et al. investigated the utilization of both NIR radiation and pH stimuli for the release of the two medicines loaded in a Janus particle for the treatment of hepatocellular carcinoma (HCC) in a study in which nanoflower-like water-soluble amphiphilic OA-UCNPs/PDA-AuF (oleic acid-NaYF4:Yb,Er/polydopamine Au nanoflower) Janus NPs loaded hydrophobic hydroxycamptothecin (HCPT) and hydrophilic DOX. After preparing polyacrylic acid (PAA) NPs, rare earth (RE) nitrate was added to create RE(OH)_3_/PAA (RP) NPs. Then, the obtained RP@PDA asymmetric NPs in the next step formed the OA-UCNPs/PDA double-layered nanobowls (NBs) using a solvothermal reaction. The OA-UCNPs/PDA NBs and HAuCl_4_ also were used to make polymer-inorganic NPs with customizable functionalities. Doxorubicin was encapsulated in the PDA compartment through π–π stacking interactions and HCPT was entrapped in the UCNP compartment by hydrophobic interactions, exhibiting loading efficiencies (LE%) of 88% and 45%, respectively. In addition, the release rates of both drugs are higher in acidic pH 5.3 than in pH 7.4, particularly HCPT at 60.5% compared to 24% in pH 7.4, which is attributed to the HCPT’s enhanced water solubility and hydrophilicity at lower pH in the JNPs [154].

Polymeric Janus particles have attracted significant interest due to the convenience with which these organic-based structures may be fabricated by polymerization, microfluidics, and solvent evaporation methods compared to their inorganic counterparts, which have shown potential for biological applications [155,156]. In 2012, the first type of Janus polymeric nanoparticle capable of dual delivery of hydrophobic and hydrophilic drugs was reported by Xie et al. [157]. To prepare the biocompatible poly (lactic-*co*-glycolic acid) (PLGA) Janus particles, the fluidic nanoprecipitation technique was utilized to create the two-faced particle in one step. The two polymers meet as they leave the two inlet streams, laid out side by side and equally exposed to the force of the dispersing phase. A uniform volume of each polymer is contained within each droplet owing to the perpendicular arrangement of the channels. Then, due to turbulence at the inlet/distribution channel interface, Janus droplets were released and solidified via the "nanoprecipitation" process to create the nanoparticles. The drugs DOX-HCl (hydrophilic) and PTX (hydrophobic), with different solubilities, were encapsulated by injecting solution A (PTX/PLGA in acetonitrile) and solution B (Dox/PLGA in 2:1 methylene chloride/methanol) into the dispersion phase (1% PVA). According to the structure of the PLGA copolymer, both medicines were effectively loaded into different portions, with 80% for PTX and 15% for DOX encapsulation efficiencies [157].

Another polymeric Janus nanoparticle PLGA/PCL was designed using double emulsion method to encapsulate various drugs, including two hydrophobic drugs, curcumin (CUR) and Quercetin (QCT), with the O/W technique. A combination of the hydrophilic drug (acetaminophen, APAP) and the hydrophobic one (naproxen, NPX) with three different emulsion methods was also performed, as below: (1) W/O emulsion with a slightly water-miscible solvent, (2) W/O emulsion with a co-solvent, and (3) W/O/W double emulsion. Single-O/W emulsion solvent evaporation encapsulated hydrophobic CUR and QCT into PLGA/PCL Janus particles. Single emulsions can encapsulate hydrophobic substances since they dissolve in the oil phase with the polymers. However, the low solubility of APAP in the oil phase necessitated modifications in the technique for single-W/O emulsions to encapsulate APAP and NPX. In the first method, ethyl acetate was used as the partially water-miscible solvent, and in the second, a mixture of DCM and methanol was employed. The third method involved the creation of a W/O/W double emulsion, which enabled the hydrophilic drug to be enclosed inside the W/O droplets as the particles’ core. The encapsulation efficiencies of the drugs CUR and QCT was analyzed and compared in the Janus and PCL nanoparticles demonstrating high encapsulation efficiency (EE%) of 93.11% for CUR and 92.03% for QCT in Janus NPs, and 93.38% for CUR and 86.9% for QCT in PCL. Moreover, the EE% of the APAP and NPX, respectively, using the indirect analysis of the supernatant was reported as follows: O/W with ethyl acetate at 54.9% and 93.98%, O/W with DCM + methanol at 21.04% and 91.88%, and W/O/W at 68.29% and 85.49%. Unlike the hydrophilic APAP medication, for which the best percentage of encapsulation effectiveness is acquired using the double emulsion technique, NPX is easily encapsulated using any method due to its superior solubility in the oil phase [158]. 

The polymeric-inorganic type of Janus structures in dual delivery systems was prepared by Khoee et al. The alkynyl-functionalized graphene oxide particle was further functionalized with azide-functionalized hydrophobic poly(ε-caprolactone) (PCL) and temperature-sensitive hydrophilic (N-isopropylacrylamide-co-acrylamide-allylamine) terpolymer on each side of the GO sheets to give the amphiphilic Janus structure using Pickering emulsions, suitable for encapsulating hydrophobic Quercetin and hydrophilic 5FU as model drugs. Regarding the high solubility of the 5FU in water and solubility of the quercetin in DMF, each nanoparticle was loaded with a different pharmacological combination, and the results were compared with those of nanoparticles carrying a single drug. Separate loading of distinct drug entities into Janus nanoparticles produces a higher EE for both pharmaceuticals than simultaneous loading. This increase is substantially significant for quercetin because it is inclined to both the hydrophilic and hydrophobic areas of nanoparticles. In addition to PCL, terpolymers with hydrophilic and hydrophobic components tend to entrap quercetin and increase its EE, while hydrophilic 5FU is loaded only in the terpolymer region. In addition, the drug sample containing solely quercetin in a Janus structure exhibited much higher toxicity in in vitro evaluations, indicating more solubility and bioavailability of the hydrophobic drug [159].

### 3.2. Anisotropic Structures Release and Interaction Data

Due to the specific and asymmetrical nature of the anisotropic particles, dual-loaded anisotropic designs may display unique characteristics. Understanding the molecular interactions between these compounds and anisotropic nanoparticle designs, such as Janus nanoparticles, may help to choose a better carrier and loading mechanism, which ultimately influences the release profile. Wang et al. (2013) reported the synthesis of a two-face superparamagnetic nanocomposite. The “Janus” structure was constructed by encasing half of a polystyrene nanoparticle (PS) in a silica shell encapsulating Fe_3_O_4_ particles, to which the anticancer drug doxorubicin was coupled to the silica layer using a dihydrizide linker, and PEG-FA was reacted with the bare surface of PS to immobilize the folic acid on the nanoparticles. One-pot miniemulsion and sol-gel synthesis were used to create superparamagnetic Janus nanocomposite (SJNCs). The PS surface was first functionalized with COOH groups and then reacted with FA-PEG-NH2 using a carbodiimide-mediated process. The immobilization of DOX on the Fe_3_O_4_@SiO_2_ surface was accomplished through imine bonding with the potential of breakage by the acidic pH of tumor-containing tissues. By selectively cleaving the linker, the receptor/drug-loaded system was stable at the neutral pH of the bloodstream, thus reducing the toxic effects of anticancer medications on normal tissue. Additionally, iron oxide nanoparticles (10 nm) were encased in a silica shell of 100 nm for use as MRI imaging agents [160] and possible application in magnetically induced hyperthermia [161]. A drug release analysis was conducted to determine the DOX release profile from FA-SJNC-DOX at two pH values. As projected by the researchers, the release rate of DOX in an acidic environment at pH 5 was 4-foldquicker than at pH 7.4 (almost 80% vs. 20%), emphasizing the desirable qualities of the systems for stability in healthy tissues. In particular, at pH 5, the overall DOX release rate was 82.6% (the trend flattened out after the burst release in the first 60 hours), whereas at pH 6, the rate was 47.1%, and at pH 7.4, the rate was 25.6%, with more gradual release slopes. The observed release rates in acidic environments suggested the breakage of the linker between DOX and carrier, which was particularly expected in the acidic pH 4.5 and 6.5 of endosomes [162].

**Figure 8:**
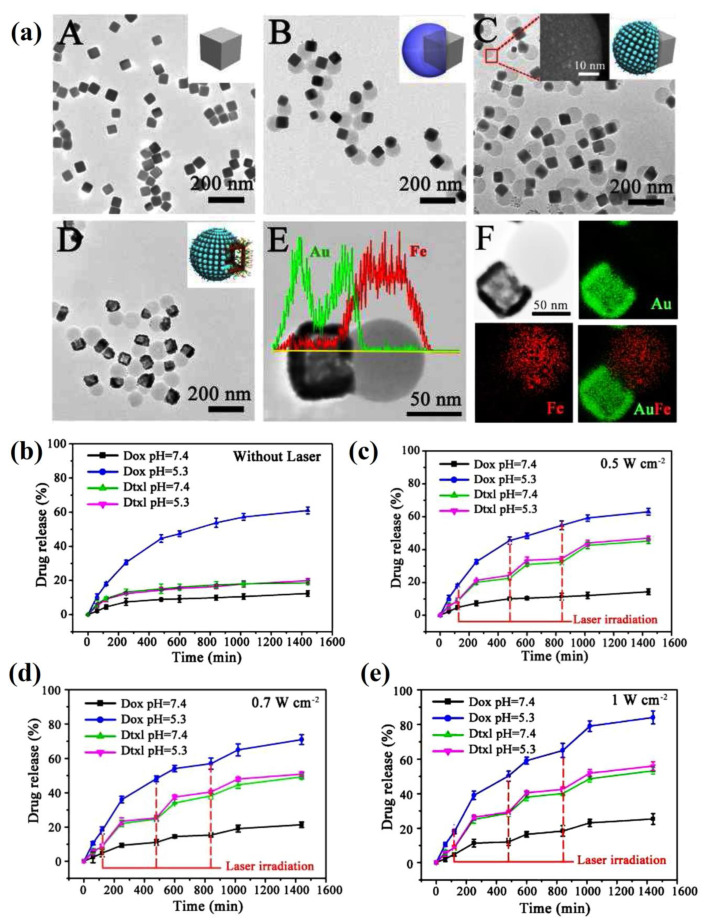
(**a**) Step-by-step synthesis AgNC (**A**), AgNC/PAA (**B**), AgNC/Fe(OH)_3_-PAA (**C**), and AuNC/Fe(OH)_3_-PAA (**D**), and images (**E**,**F**) are the proof of the asymmetrical structure of nanoparticles by elemental line scan and elemental mapping. (**b**–**e**) Release patterns of Dox and Dtxl at various time intervals in PBS (pH = 5.3 and 7.4) under and without NIR irradiation with various intensities (0.5, 0.7, and 1 W cm^−2^, for 5 min). Reproduced from Zhang et al. [165], with permission from Elsevier.

Janus nanoparticles, which took the form of unique cuboid-shaped spheres, were designed for simultaneous or sequential drug administration, imaging with CT and MRI, and susceptibility to NIR radiation. Ag nanocubes (AgNCs) were mixed with PAA, and then, by shifts in interfacial energy, the precursors spherical/cubic Ag/PAA JNPs were created. Selective development of Fe(OH)_3_ on the PAA side then provided the resulting Fe(OH)_3_-PAA portion, pH stimuli-responsivity, MR imaging, and hydrophilic drug loading capabilities. Notably, PAA’s network used its preserved water during this stage to hydrolyze Fe^2+^ into Fe(OH)_3_. Meanwhile, HAuCl_4_ etched AgNC to generate AuNC, resulting in a hollow structure well suited to the administration of hydrophobic drugs. The AuNC component of the AuNC/Fe(OH)_3_-PAA JNPs was then modified with PCL-SH, offering the carrier NIR stimuli-responsive features, CT imaging, hydrophobic drug storage (Figure 8). Additionally, the nanoparticles were loaded with docetaxel drug abbreviated as Dtxl (hydrophobic drug) at a loading capacity of 5 wt%, followed by DOX (hydrophilic drug) at 20 wt% in hthe Fe(OH)_3_-PAA portion via electrostatic interaction between positively charged DOX and negatively charged PAA. Drug release profiles in the sequential and simultaneous releases were assessed using pH and NIR as effective factors. The very identical release patterns of Dtxl in neutral and acidic PBS suggest that the hydrophobic drug requires a long time (perhaps days) to reach a high release rate at physiological pH, due to its hydrophobicity and interactions with JNPs. Up to 4 fold the release of DOX at an acidic pH compared to the negligible release of DOX in neutral PBS is due to the pH-sensitivity of PAA and instability of Fe(OH)_3_ NPs in acidic conditions. Upon application of a pH stimulus, DOX can diffuse from the degraded Fe(OH)_3_-PAA portion in a burst release manner. When the effect of irradiation on release was examined, NIR laser irradiation at 0.5 W cm^−2^ for 5 min enhanced the release of Dtxl at pHs 7.4 and 5.3; however, the release profiles of DOX at pH 7.4 and pH 5.3 were almost not impacted (Figure 8). At both pHs, increases in the total quantity of Dtxl released were observed when higher NIR laser power densities were implemented (0.7 W cm^−2^ and 1 W cm^−2^, 5 min). Changes in temperature as a consequence of NIR irradiation and their effects on release alteration demonstrated that the lower intensity of NIR irradiation only impacted AuNC by raising its temperature, which decreased the viscosity of Dtxl and facilitated its detachment from SH-PCL [163,164], while Fe(OH)_3_-PAA parts containing DOX were hardly affected. When the irradiation intensity is raised, more heat is introduced into the PCL and Fe(OH)_3_-PAA sections resulting in the release of both medicines. In this regard, pH and NIR irradiation can be used to regulate the sequential and simultaneous release of medications from the same Janus structure [165].

A nanocomposite of Janus mesoporous silica nanoparticles with two distinct compartments was developed for use in research on dual delivery systems. Through anisotropic island nucleation and growth, cubic-sphere-shaped porous UCNP@SiO_2_@mSiO_2_&PMO (UCNP or upconversion NPs: NaGdF_4_:Yb, Tm@NaGdF_4_, PMO = periodic mesoporous organosilica) nanocomposites sensitive to UV/Vis irradiation and heat induced by NIR irradiation were synthesized. During the synthesis procedure, a 25-nm-dense coating of silica was deposited on the hydrophobic surface of the UCNP core to form the core–shell UCNP@silica nanoparticle, which was then ready for usage in the subsequent step. Furthermore, the UCNP@SiO_2_ was coated with a 30 nm ordered mesoporous silica shell, and cubic PMO nanoparticles were produced on one side of the spherical nanoparticles using CTAB as templates and 1,2-bis(triethoxysilyl)ethane (BTEE) as a silane precursor. Moreover, since there was no lattice-match relation between the channels of the mesoporous silica and the cubic PMO, the formation of the single-crystal PMO on the UNCP@SiO_2_@mSiO_2_ occurred at random, resulting in an island of PMO. Mesoporous channels were functionalized with a light-sensitive azobenzene molecule, and then the exterior surface of the particles was coated with 1-tetradecanol with heat-sensitivity (phase change material, PCM) on to examine the loading and release properties of the resultant material. Any chemical encapsulated in the PMO region of this structure will be released at temperatures over the PCM melting point. Further, UCNP compounds functionalized with azo groups will undergo a cis-to-trans and trans-to-cis transition under UV and visible irradiation, respectively. When NIR is induced, photons emitted by the UPCN are absorbed by azobenzene molecules, creating a propelling mechanism that aids any encapsulated substance to release. The effect of these parameters was examined further by incubating PMO hydrophobic section containing the two drugs DOX and paclitaxel with Hella cells to investigate the outcome of stimulus-induced drug release on cells. Compared to no-stimulating systems, the dual-drug-loaded UCNP@SiO_2_@mSiO_2_-Azo and PMO-PCM Janus nanocomposites significantly improved cancer cells cytotoxicity (25% killing efficacy) When subjected to heat treatment (39 °C). As a result of PCM melting (the first switch), the paclitaxel molecules are released from the mesopores of PMO. Moreover, paclitaxel and DOX molecules can be released concurrently by simultaneous use of heat and NIR light and increase cytotoxicity [166]. 

**Figure 9:**
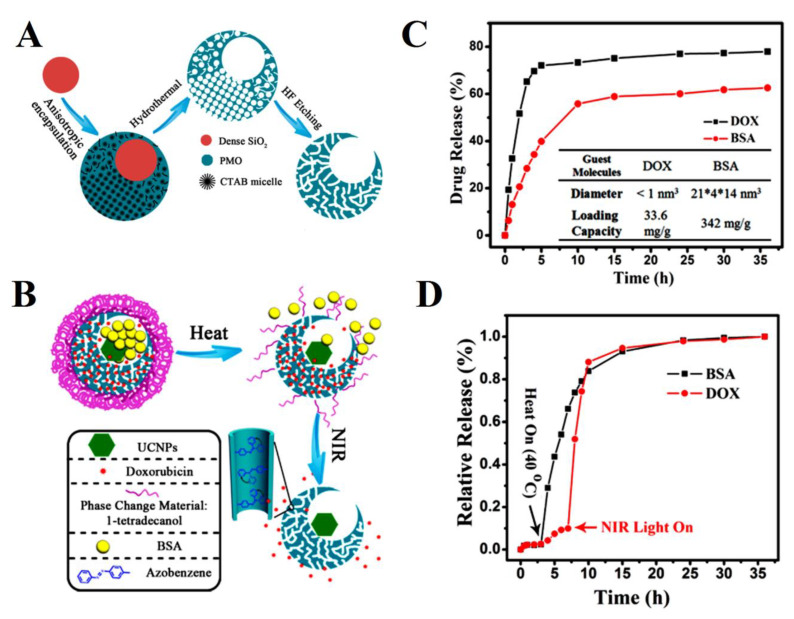
(**A**) Single-hole anisotropic mesoporous silica nanoparticle synthesis schematics. (**B**) Dual-loaded nanoparticle release mechanism through induction of heat and NIR irradiation. (**C**) Bare DOX and BSA release from carriers without stimuli. (**D**) The usage of heat and light stimuli impacts BSA and DOX release profiles. Reproduced from LI et al. [167]. Copyright © 2015, American Chemical Society.

An anisotropic single-hole structure was constructed for storing large molecules in the structure’s single hole using the same material as in the previous study; smaller holes on the exterior surface could encapsulate smaller molecules. Dense SiO_2_ coated on the UPCN was employed as the initial seed for the carriers, which were then covered by PMO using CTAB as the surfactant and BTEE as the silica precursor. An asymmetrically hollow PMO nanoparticle was then formed by etching the dense SiO_2_ using a hydrothermal process. Finally, in order to make an open PMO hole containing UPCN, the PMO shell was etched with HF solution. Since the final structure was comprised of two storage areas, bovine serum albumin (BSA) and DOX were chosen as the larger and smaller guests, respectively. The stimuli-free setting for the release of loaded guests revealed that the DOX release trend reached a plateau after 5 h at nearly 70%, while the BSA release trend leveled out after 15 h, most likely due to their distinct sizes and storage regions. Similarly, the light and heat sensitivities of this structure were generated from the azobenzene molecules in the mesopores, and the 1-tetradecanol (PCM) on the exterior shell. Notably, above the PCM melting point, BSA trapped in the hole rapidly escaped the structure owing to the outer layer melting. When exposed to UV light (360 nm), trans-isomer Azo molecules underwent a photochemical transition to a cis-isomer, while when exposed to visible light (450 nm), that cis-isomer converted into a trans-isomer (Figure 9). Moreover, the photons emitted by the UCNPs exposed to NIR light (980 nm) were readily absorbed by the Azo molecules in the mesoporous shells, creating a continuous propelling motion and aiding the release of DOX encapsulated in the shell. Overall, the comparison of release profiles in the absence and presence of stimuli, highlights the release dependency and control properties, NIR irradiation and heat at 40 °C showed the effect of both PMC melting (first release control switch) and the azo molecule influence on release (second release control switch). The significant release of DOX and BSA highlighted the dependency and control of the release by UV/NIR and heat as stimuli [167]. 

Cheng et al. constructed anisotropic lollipop-shaped nanoparticles of Fe_3_O_4_@SiO_2_&EPMO for an antibacterial effect study in 2019, where EPMO refers to ethane-bridged periodic mesoporous organosilica. Curcumin (CUR), a hydrophobic molecule, and gentamicin sulfate (GS), a hydrophilic antibiotic, were the two drugs utilized in the antibacterial treatment. The lollipop shape was formed by synthesizing the EPMO nanorod randomly on one side of core–shell Fe_3_O_4_@SiO_2_ nanospheres through a nucleation and growth process, using BTEE as the organosilica precursor. Regarding the loading of two drugs, the loading capacity of GS at 249 mg g^−1^ was higher than that of the CUR at 25.8 mg g^−1^ due to the hydrogen bond formation between the amine and hydroxyl groups in the GS molecular structure with the silanols (Si-OH) of the mesoporous silica; while CUR was attracted to the EPMO via hydrophobic–hydrophobic interactions. Further, the release profiles of the drugs were evaluated in Fe_3_O_4_@SiO_2_-GS&EPMO, Fe_3_O_4_@SiO_2_&EPMO-CUR, Fe_3_O_4_@SiO_2_-GS&EPMO-CUR systems, where GS displayed a burst release of nearly 75% in the first 2 h in the both of the GS containing systems and reached to 99.2% in Fe_3_O_4_@SiO_2_-GS&EPMO and 96.8% in Fe_3_O_4_@SiO_2_-GS&EPMO-CUR after 30 h. Nevertheless, the release rate of CUR showed a slower trend from Fe_3_O_4_@SiO_2_&EPMO-CUR at 63% and Fe_3_O_4_@SiO_2_-GS&EPMO-CUR at 50.3% after the same duration. Notably, the release of each drug had a negligible effect on the other, indicating the independent release mechanisms [168]. 

Since nanoparticle–carrier combinations of DOX and PTX were effective against a variety of cancers in several studies [169,170], Xing et al. designed a pH and NIR-responsive Janus nanoparticle composed of gold and mesoporous silica for the simultaneous administration of these drugs. The Pickering emulsion technique was employed to create the nanoparticles, and the final Janus structure was the result of coating approximately half of the MSN-NH_2_ trapped in wax by Au. Additionally, carboxylic acid groups were created on the MSN side through succinic anhydride, reacting with DOX (58.05% loading efficiency), and PTX was connected to the gold surface previously functionalized with thiol-β-cyclodextrin (SH-β-CD) (95.23% loading efficiency). To evaluate the stimuli-responsive release of the drugs, drug-loaded particles were investigated at two pH values, 7.4 and 5, as well as 5 min of NIR laser irradiation at 2.0 W/cm^2^. PTX release from nanoparticles appeared indifferent to variations in pH (almost 63%), in contrast to pH-sensitive DOX, whose release rate enhanced in acidic PBS (pH 5) by 51.17%, from 22.38% release rate at pH 7.4. Nevertheless, NIR laser conditions showed a gradual increase in the amount of PTX released at 67.55% (pH 7.4). This boost in the release rate of PTX may be attributed to the cleavage of the Au-S bond, which liberates the PTX–CD component, which is further broken by NIR laser heating, resulting in a cyclodextrin-free PTX drug. The release of DOX, when subjected to NIR irradiation, on the other hand, was unaffected by the irradiation and exhibited relatively similar release patterns and rates to settings with pH value variation [153]. 

Real-time monitoring of multiple drugs from one carrier is an important issue in drug delivery systems since most of the studied real-time analyses were assigned to single-drug systems. The obstacles of intercellular real-time monitoring of multiple medications, such as the need for independent drug release analysis for each drug led to the development of a Janus nanoparticle of DOX-CMR-MS/Au-6MP (DOX: doxorubicin, CMR: 7-hydroxycoumarin-3-carboxylate, MS: mesoporous silica, Au: gold, 6-mercaptopurine), in which FRET and SERS were used for real-time monitoring the release of two drugs DOX and 6MP. The fluorescence resonance energy transfer (FRET) mechanism, wherein energy is transmitted from a fluorophore donor to another chromophore or acceptor, has been utilized as an imaging tool for the real-time monitoring of drug release [171]. Surface-enhanced Raman scattering (SERS) also amplifies the Raman signals of immobilized materials on gold or silver surfaces [172]. CMR-incorporated MS was first produced using co-condensation and sol-gel techniques then the 6MP-containing Au nanoparticles were coated on half of the nanoparticles in the paraffin obtained from the Pickering emulsion to form the final Janus structure. To accomplish this, 6MP was adsorbed to the Au surface via Au-S interactions, while the nanochannels of CMR (FRET donor) were attached to DOX (FRET acceptor) via a pH-responsive linker hydrazone which was expected to be cleaved at the endosomal pH of 5–6 or via GSH (glutathione ethyl ester) trigger. As a significant reducing agent in biological processes, glutathione (GSH) has been employed as an in situ releasing reagent in live cells [173]. For example, AuNP surfaces were subjected to GSH-mediated in vitro release of the fluorescent thiolated dye [174]. The DOX release from nanoparticles was monitored in real time after an effective FRET between DOX and CMR occurred. The UV-Vis absorption spectra of DOX and the fluorescence emission spectra of CMR demonstrated that the excited CMR at 405 nm acted as a photon donor to DOX with maximum absorption at 480 nm, where while the weak initial intensity of CMR increased considerably over time, the UV intensity of DOX (λ_em_ = 595 nm) decreased. Additionally, the CMR fluorescence spectra in PBS at pH 5 revealed that the intensity changed minimally in the absence of DOX, indicating the stability of CMR at an acidic pH, and most importantly, the rise in CMR intensity in the NPs containing DOX was entirely attributable to the release of DOX and the reduction in FRET efficiency. The 6MP release was monitored via SERS analysis using GSH as well. Raman spectra displayed no response to free 6MP, DOX, or GSH; however, DOX-CMR-MS/Au-6MP incubated with GSH (for 1 h) exhibited a decreasing intensity as GSH content increased. The duration of 6MP incubation in 5 mM GSH reduces the SERS signal intensity. After incubation with 5 mM of GSH, there was no change in the Raman spectra of Janus DOX/NPs, confirming that the reduction in SERS intensity of incubated Janus DOX/6MP NPs was related solely to the release of 6MP. In this regard, both FRET and SERS could be feasible in the real-time release monitoring of two drugs. In addition, research into the pH-sensitive release of drugs demonstrated that the release rate of DOX was substantially higher in acidic pH than in neutral pH (60% at pH 5 vs. 15% at pH 7.4), which points to the durability of the hydrazone bond at pH 7.4 and its cleavability in acidic conditions [175].

It has been proposed that in addition to Janus nanoparticles, anisotropic structures with multicompartment and patchy surfaces might incorporate pharmaceuticals or chemical components, thus providing viable frameworks to be utilized in drug delivery systems [176]. They are promising carriers in dual delivery systems, such as multicompartment micelles, because of their unique ability to take up compounds with varied characteristics within their numerous compartments [177,178]. A temperature-sensitive multicompartment hydrogel (MCH) was developed for the co-encapsulation of paclitaxel and doxorubicin, while its distinctive compartment formation during the assembly process was evaluated via fluorescence resonance energy transfer (FRET). Two amphiphilic triblock copolymers, poly(ε-caprolactone-co-1,4,8 trioxa [4.6]spiro-9-undecanone) -poly(ethylene glycol) -poly(-caprolactone-co-1,4,8-trioxa[4.6]spiro-9-undecanone) (PECT) and mPEG-poly(2 (perfluoro butyl)ethyl methacrylate) (PPFEMA) assembled cooperatively in water to form MCH nanoparticles. Due to their amphiphilic features, both copolymers could encapsulate drugs in their hydrophobic cores. PECT was loaded with PTX with 0.75% (*w*/*w*) drug loading amount, while DOX was encapsulated in PPFEMA at 4.2% (*w*/*w*). The two drug-loaded particles were further integrated via an increase in the temperature to give the eventual MCH. Comparing the release of PTX-loaded MCH and DOX-loaded MCH nanoparticles separately and together allowed the examination of drug release rates and drug–drug interaction and its influence on release patterns. The rate of PTX release from solely PTX-loaded MCH was 62.2%, whereas the rate of PTX release from MCH containing both medicines was 64.9%. 20.7% of the DOX-loaded MCH and nearly 23% of the dual-loaded system exhibited sustained release of DOX over 42 days. These results highlight the exclusive mechanisms of drug release and the lack of drug–drug interferences because the diffusion rates of the two drugs were not affected by each other even though they were both released from the same carrier, MCH. This is especially true for the release of DOX, which had a diffusion release mechanism due to the slow degradation of the fluorocarbon segment. The resulting formulation would allow for the sustained, localized administration of any drug within a predetermined concentration range [179].

**Figure 10:**
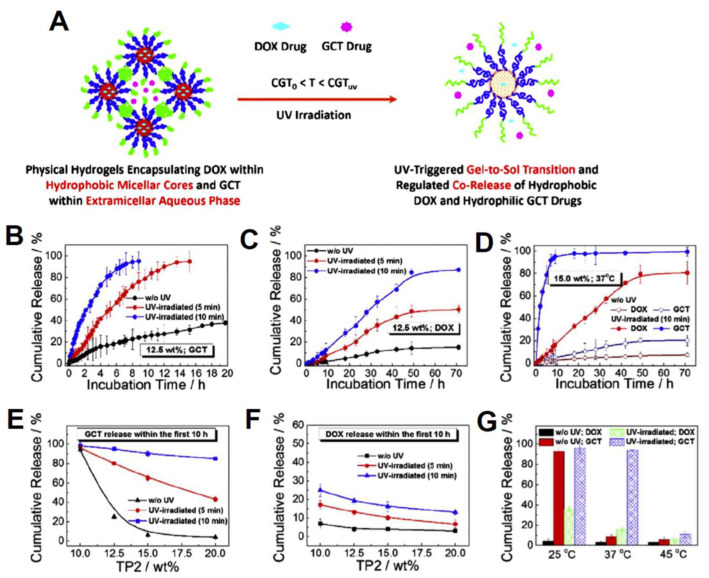
Temperature and UV irradiation affect the drug release profiles of DOX and GCT. (**A**) A schematic illustrating the UV-induced gel-to-sol transition and thermal drug release from TP2 hydrogels containing DOX encapsulated in hydrophobic micellar centers and GCT in the extra micellar aqueous media. (**B**) GCT release profile with and without UV irradiation. (**C**) DOX release profile with and without UV irradiation. (**D**) A comparison of DOX and GCT release profiles after 10 min of UV irradiation to DOX/GCT co-loaded TP2 hydrogels (15.0 wt. %) at 37 °C. (**E**) GCT cumulative release from TP2 hydrogels with and without UV irradiation in the first 10 h. (**F**) DOX cumulative release from TP2 hydrogels with and without UV irradiation in the first 10 h. (**G**) Comparison of DOX and GCT cumulative release from with and without UV irradiation (10 min) from TP2 hydrogels (15.0 wt. %; pH 7.4 buffer) throughout the first 10 h with temperature as the main alternative. Reproduced from Wang et al. [180], with permission from Elsevier.

Use of copolymers to assemble into amphiphilic carriers for co-delivery of drugs was also investigated by Wang et al. to synthesize a thermos and UV-sensitive multicompartment hydrogel to encapsulate hydrophobic DOX and hydrophilic gemcitabine (GCT). The triblock copolymer, poly(*N*-isopropylacrylamide)-b-poly(4-acryloylmorpholine)-b-poly(2-((((2-nitrobenzyl) oxy) carbonyl) amino) ethyl methacrylate) (PNIPAM-b-PNAM-b PNBOC), synthesized via RAFT polymerizations steps, were assembled into micellar nanoparticles with cores of photo-responsive PNBOC, inner shells of hydrophilic PNAM, and coronas of thermo-responsive PNIPAM at temperatures below lower critical solution temperature (LCST) at a relatively low concentration. Notably, to evaluate the ability of triblock polymers to generate hydrogels, the triblock copolymers were dissolved in PBS buffer (pH 7.4; 10 mM) at four different concentrations of 10.0, 12.5, 15.0, and 20.0 wt. %. When the polymer concentration increased above 10.0 wt. % at room temperature, opaque sol solutions were formed, which gelled upon heating beyond the critical gelation temperature (CGT_0_) of triblock copolymers. CGT (CGT_UV_) of irradiated triblock copolymers is boosted when NBOC moieties create primary amine groups in the presence of UV radiation, triggering a cascade of reactions that cross-link nanoassemblies and concurrently render their cores hydrophilic. Thus, the irradiated sol solutions transformed into hydrogels at elevated temperatures, and the gel-to-sol transition occurred at CGT_0_ < T < CGT_UV_. Due to the formation of micellar networks at 37 °C and 20 wt. %, the release profile of DOX and GCT from hydrogels containing 34 repeating units of PNBOC in the copolymer chain (TP2) was studied, where DOX and GCT were encapsulated inside the micellar cores and extramicellar aqueous phase, respectively (Figure 10). Before UV irradiation, slow release of both drugs was observed, specifically slower for DOX as a result of its entrapment in the micellar cores and taking a longer time to release. Although DOX was still released at a slower rate than GCT after UV irradiation, the release rates of both medications were increased compared to the those without UV irradiation due to the gel-to-sol and hydrophobic-to-hydrophilic transitions. The effect of different drug loading sites was evident in the delayed release of DOX, each of which independently affected the medications’ discharge rates. When the concentration was reduced to 10 wt. %, the diffusion of GCT out of the micelles became uncontrolled because of the gel-to-sol transition; however, the slightly quicker release of DOX could still be modulated by UV irradiation. The 12.5 wt. % concentration of hydrogels under UV irradiation was also used to study the co-release of both medicines. Again, in the presence of UV irradiation at a fixed concentration of hydrogels, enhanced release of both drugs was observed, whereas increased concentration resulted in reduced release rates of drugs. Furthermore, the effect of temperature on the release profiles of both drugs was studied at 15 wt. % and 37 °C, where after 70 h, less than 20% GCT and 10% DOX were released without UV. Higher release rates under UV irradiation at that temperature were due to the gel-to-sol transitions, similar to the results obtained from other concentration of 12.5 wt. % and 20 wt. %. Reduced temperature (25 °C) caused GCT to diffuse into the aqueous phase, demonstrating that its release rate was independent of UV irradiation, but UV irradiation correlated with the DOX release profile. This was observed when both drugs were introduced into the hydrogel solution. Nevertheless, the temperature rises of the hydrogel solution up to 45 °C prevented the gel-to-sol transition following UV irradiation, but a hydrophobic-to-hydrophilic transition still occurred. Together, the release of DOX and GCT can be adjusted by UV light irradiation and changes in temperature [180].

**Figure 11:**
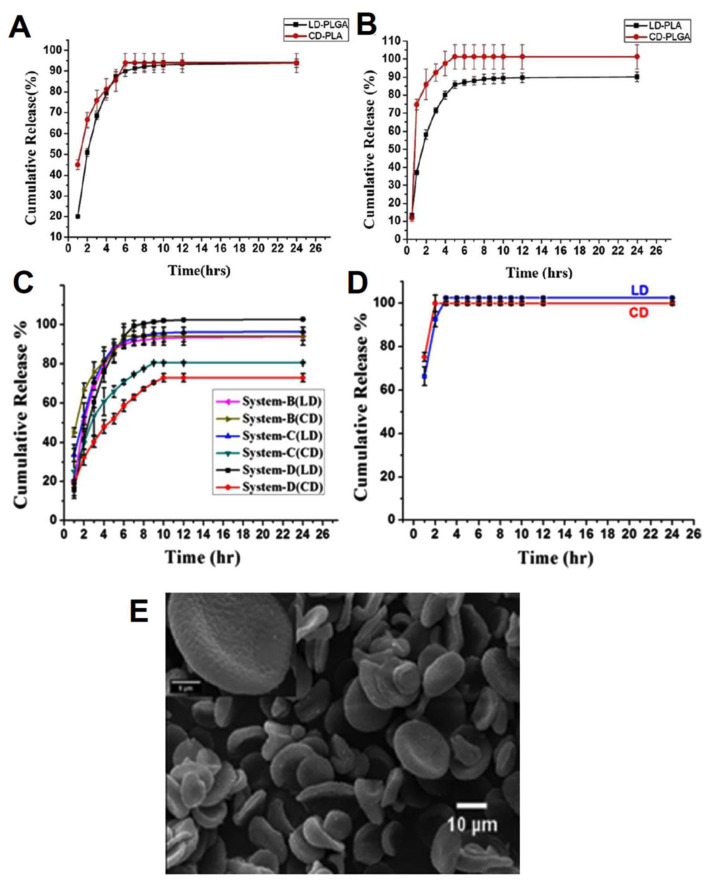
LD and CD release profiles from (**A**) system A and (**B**) system B. (**C**) LD and CD release rate comparison in systems B, C, and D. (**D**) The dual-drug release from the Syndopa tablets in SGF for 5 h + 24 h in SIF. (**E**) SEM analysis pictures of the disc-shaped dual-compartment materials. Reproduced from Parthipan et al. [187], with permission from Springer Nature.

Combinations of the prodrug levodopa (LP) and the inhibitor of the AADC enzyme decarboxylating LP, carbidopa (CD), have been found to be beneficial for the treatment of Parkinson’s disease (PD) [181]. By using CD in the pharmacological combination, the bioavailability and efficacy of LD might be improved, producing the dopamine essential for the PD treatment at a lower dosage with fewer side effects [182]. However, despite the synergism of LD and CD in delivering LD to the brain, the drugs’ short half-lives render it challenging to administer LD continuously, even after several doses, to maintain oral plasma bioavailability [183]. Therefore, disk-shaped dual-compartment nanomaterial of amorphous poly(lactide-*co*-glycolide) (PLGA) and semicrystalline polylactide (PLA) were designed using the electrohydrodynamic co-jetting (EHDC) technique for addressing the challenges of co-administration of the LD/CD and the release investigation in simulated gastric (SGF) and intestinal fluid (SIF). EHDC is a simplistic and adaptable method to fabricate multicompartmental micro- and nanoparticles in different geometries, including discs, rods, and cylinders [184,185,186]. The final disc-shaped material was obtained from a 4.5% *w*/*w* solution of both PLGA and PLA and a stable jet at a flow rate of 1.5 mL/h. It is likely that at higher polymer concentrations, the layer developed at the outer surface of the material resulting from quick solvent evaporation, slowly lost its focal solvent, followed by hollow spherical particles collapse, leaving a disc-shaped anisotropic nanoparticle with a rougher surface on the PLA side, owing to its semicrystalline nature (Figure 11). The change in the molecular weight or crystallinity of the polymers could ultimately affect the anisotropicity of the particles’ structure. However, due to the restrictions at shrinkage posed by loaded drugs in the polymers, the nanoparticles containing drugs somewhat displayed roughness at both compartments. Considering that the stomach and upper intestine were the primary sites of absorption for these medications, a release study of dual drug-loaded particles was carried out in SGF for 5 h and then in SIF, assuming that particles would remain in the stomach for 5 h before entering the intestinal tract. Two systems, A (LD in PLA and CD in PLGA) and B (LD in PLGA and CD in PLA), were chosen for the initial release analysis. The release of both LD and CD from the PLA compartment was much slower than that of the PLGA compartment due to the semicrystalline morphology and more hydrophobic nature of PLA, which may have retarded the hydrolytic degradation and possibly reduced the rate of drug release [187].

Moreover, despite the 4-fold higher concentration of LD in comparison with CD, its release rate is slower than CD from PLGA, indicating the better administration of drugs in systems B with LD loaded in PLGA and CD in PLA. The release profiles of system B illustrated nearly 90% of drugs were released after 24 h (Figure 11). Additionally, two more systems, C and D, were tested with a 4:1 (LD:CD) drug content to explore different drug formulations. Evidently, despite the same burst release pattern of pharmaceuticals similar to system B under the same circumstances, the CD release rate reduced as the content increased, probably due to the improved interaction of CD at higher content with PLA, which resulted in lower release rates. However, the poor interaction between the LD and PLGA and the facilitation of water penetration into the polymer by increased drug concentration led to nearly unchanged LD release rates. In addition, a release study of both medications was conducted to investigate the dual release of drugs from the commercial Syndopa tablet with a fixed combination of LD and CD, where a 100% release of drugs was observed within 2 h without any regulated and sustained drug release. To reach the needed daily controlled dosage, the developed drug delivery system might be advantageous, particularly sample B, which had the most effective release profile [187].

### 3.3. Internalization and Cytotoxicity Evaluations

Since there has been little research with a specific focus on the influence of the anisotropic surface of particles on their entrance into cells or how it may be used to regulate their cytotoxicity and biomedical function, reviewing these factors in recent dual-delivery-associated studies may provide crucial data on medical treatments utilizing anisotropically structured particles. Functionalization of the surface with targeting ligands, aptamers, proteins [188], and PEGylation [189,190] are methods to facilitate the internalization and targeting ability of the materials inside the cells. For example, dual-functionalized Janus nanoparticles of HA-JMSN/DOX-DMMA, where anionic HA (hyaluronic acid) functions as an affinity agent with CD44 receptors of cells, and DMMA (2,3 dimethylmaleic anhydride) cleaves as a result of acidic pH, resulted in improved cell uptake of Janus materials for the administration of single-drug doxorubicin. The cytotoxicity, cell uptake, and DOX delivery into the cells of Janus nanoparticles were higher than their isotropic/uniform counterparts. It confirmed the active targeting capability of the HA ligand, since cell uptake of HA-JMSN/ DOX-DMMA was significantly decreased following inhibition of the CD44 receptor on A549 cells by free HA. The efficacy of HA-JMSN/DOX-DMMA in targeting tumors was validated in animal tests. Mice with tumors treated with HA JMSN/DOX-DMMA grew much fewer tumors than mice treated with isotropic nanoparticles, suggesting the potential efficacy of the dual-functionalized Janus nanomaterial in tumor treatments [191]. The enhanced targetability in anisotropic designs was also studied by Gao et al., where serum bovine albumin (BSA) was formed into a sphere and functionalized with indocyanine green-amine (ICG-NH2) after the deposition of mono-AuNPs on the surface of BSA NPs, through an adsorption-reduction seed-mediated growth. The bare surface of AuNPs was functionalized with AS1411-thiol aptamer to form the snowman structure. DOX, a model drug, was also loaded on BSA with 62% loading efficiency. The tumor volume of mice treated with AS1411-AuBJNP-ICG/DOX while exposed to 808 nm NIR laser irradiation was shown to be significantly decreased by in vivo examination. Tumor suppression was also higher in the AS1411-AuBJNP-ICG/DOX group without NIR laser irradiation compared to the free DNA control, revealing that aptamers can improve nanomaterials’ targeting. These multifunctional T2-phage-like AS1411-AuBJNP-ICG nanomaterials provided a biomimetic system suitable for active cancer targeting and chemo-photothermal treatment [192].

A combined drug-delivery of doxorubicin and berberine (BER) utilizing Janus magnetic mesoporous silica nanoparticles was also investigated, employing HA as a targeting agent that enables cells to absorb HA-modified nanocarriers via the CD44 receptor-mediated pathway. In this study, the regeneration of cancer cells in hepatocellular carcinoma (HCC) associated with calcium-independent phospholipase A2 (iPLA2) [193,194] was investigated using BER as the iPLA2 inhibitor [195], and DOX against this “Phoenix Rising”. As in vivo and in vitro studies demonstrated the effect of BER on suppressing the repopulation of cancer cells induced by DOX, the drugs were loaded into nanoparticles whose structure was formed through the use of the sol-gel technique, followed by HA functionalization on the silica surface (loading efficiencies: DOX at 58.81% and BER at 54.17%). As a first step in evaluating the HCC targeting effect of HA-MSN@DB, confocal laser scanning microscopy (CLSM) was used to observe the uptake of FITC-labeled HA-MSNs on CD44 receptor-overexpressing HepG2 cells. The endo/lysosomal system was the location of both MSNs and HA-MSNs after they were taken up by cells. The CD44 receptor selectivity features of HA-MSNs were also proven in H22, NIH-3T3, and HL-7702 cell lines using flow cytometry. Cell viability and cytotoxicity evaluation were further performed using HepG2, HL-7702, H22, and NIH-3T3 cells by five systems of MSNs, DOX, DOX+BER, MSN@DB, and HA-MSN@DB. HepG2 and HL-7702 cells were dose dependently inhibited by MSN@DB and HA-MSN@DB, whereas HA-MSN@DB with DOX and BER at equal concentrations displayed a much higher anticancer efficacy than MSN@DB. While BER did not independently impair cell viability even at the highest dose, a certain amount of BER was observed in HepG2 cells. Moreover, the results of flow cytometry demonstrated the highest apoptotic efficiency of HA-MASN@DB at 48.10% among other systems, in agreement with cell viability results. Further, the anti-reoccurrence and repopulation of cancer cells in mice with the same sample groups showed that while the inhibition of tumor growth was successful in the first 7 days, the reoccurrence after chemotherapy was observed in mice treated with DOX. Nevertheless, the HA-MSN@DB and BER+DOX had considerable growth prevention in addition to their ability to prolong the survival of mice, with HA-MSN@DB owning the best performance among other groups. Through the use of MRI and ICP-OES for Fe element detection in mouse organs, the effect of HA-MSN@DB on tumor targeting in H22 malignant cells in mice was analyzed. Both techniques showed that MSN@DB and HA-MSN@DB concentrated in tumor areas, with HA-MSN@DB demonstrating a higher tumor-site concentration due to its CD44 receptor-mediated targeting capability [196].

**Figure 12:**
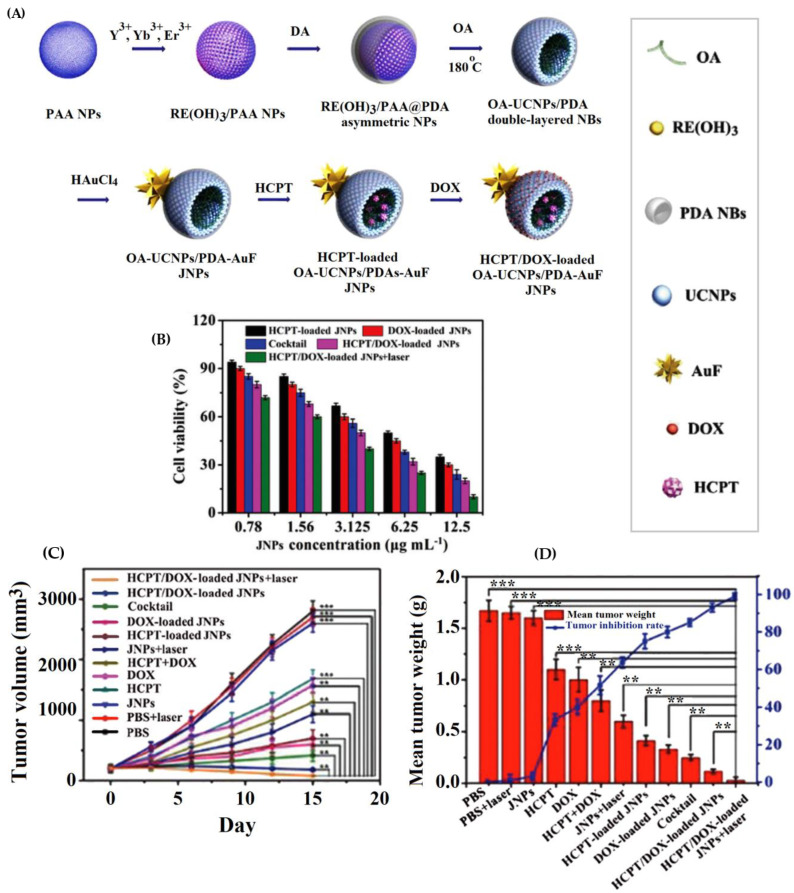
(**A**) Schematic of HCPT/DOX-loaded OA-UCNPs/PDA-AuF JNP synthesis process. (**B**) Cell viability results of test groups attained from MTT assay. (**C**) Tumor growth patterns per day. (**D**) Tumor weight inhibition rate from each test group (** *p* < 0.01 and *** *p* < 0.001). Reproduced from Chen et al. [154], with permission from the Royal Society of Chemistry, Great Britain.

Additionally, the data from the previously modified 2021 research by Chen et al. reported a further approach to treating HCC, which used pH and NIR laser as stimuli for dual-drug-loaded oleic acid-NaYF4,Yb,Er/polydopamine Au nanoflower JNPs [154]. As mentioned earlier, DOX and HCPT were used as model drugs loaded on the PDA and UCPA, respectively. Using the standard MTT test, the PTT impact of OA-UCNPs/PDA-AuF JNPs was determined, indicating a significant reduction in HepG2 cell viability when incubated with OA-UCNPs/PDA-AuF JNPs induced by laser irradiation (10 min, 1 W/cm^2^), whereas the group without laser irradiation demonstrated no toxicity. Additionally, a combination index (CI) study (CI 1, =1, or >1, respectively, as synergism, addition, and antagonism effects) utilizing HepG2 cells showed that HCPT/DOX-loaded OA-UCNPs/PDA-AuF JNPs with 1:1 drugs’ molar ratio demonstrated the lowest CI values and the highest synergistic impact, consistently outperforming the cocktail system. It was demonstrated by the fact that the HCPT/DOX-loaded OA-UCNPs/PDA-AuF JNPs could produce a more uniform drug release than the cocktail system, therefore enhancing the synergistic impact of the combination. Furthermore, OA-UCNPs/PDA-AuF JNPs displayed superior inhibitory effect and cytotoxicity towards HepG2 cells among all other tested groups (Figure 12). More importantly, CLSM pictures showed that the HCPT/DOX-loaded OA-UCNPs/ PDA-AuF JNPs exhibited time-dependent patterns for absorption and release within the cell, further demonstrating that the HCPT and DOX molecules were co-delivered and subsequently aggregated in the nucleus upon passing the nuclear membrane. However, laser irradiation of tumor cells loaded with HCPT/DOX in OA-UCNPs/PDA-AuF JNPs demonstrated a 99% inhibition of cell growth, with a higher inhibition rate in the single-drug loaded JNPs compared to the free drug groups through in vivo tests, further validating the significant performance of dual-drug JNPs and enhanced accumulation of JNP groups in tumor sites [154].

To implement photothermal therapy (PTT), Li et al. fabricated zeolitic imidazolate framework-8 (ZIF-8)/PDA hollow Janus nanoparticles decorated with heptakis-(6-mercapto-6-deoxy)—cyclodextrin (CD) on the PDA side and loading it with hydrophobic drug 10-hydroxycamptothecin (HCPT), while loading the ZIF-8 cavities with hydrochloride doxorubicin (DOX). In addition, the nanoparticles were functionalized with PEG to increase their solubility. The electrostatic interaction between the electronegative carboxyl groups of PAA and the electropositive DOX enables the hydrophilic DOX to be loaded into the ZIF-8 domain, while the hydrophobic HCPT could be trapped within the hydrophobic cavities of CDs coupled to PDA surfaces, showing drug loading capacities of 42.0 wt% for DOX and 9.8 wt% for HCPT. The CCK-8 assay on HepG-2, HeLa, and MCF-7 cells, resulted in cell viabilities of 91.3%, 92%, and 90.9% for HepG-2, HeLa, and MCF-7 cells, respectively, in addition to the hemolysis test showing excellent biocompatibility (2.32% at the highest concentration) were conducted to evaluate the potential cytotoxicity and biocompatibility properties of the H-ZIF-8/PDA-CD JNPs, respectively. Furthermore, HepG-2 cells treated by HCPT-loaded H-ZIF-8/PDA-CD JNPs, DOX-loaded H-ZIF-8/PDA-CD JNPs, HCPT-loaded H-ZIF-8/PDA-CD JNPs mixed with DOX-loaded H-ZIF-8/PDA-CD JNPs (cocktail), H-ZIF-8/PDA-CD JNPs plus laser, DOX and HCPT co-loaded H-ZIF-8/PDA-CD JNPs, DOX and HCPT co-loaded H-ZIF-8/PDA-CD JNPs plus laser were used for the analysis of cell viability and evaluation of PTT, where results suggested that co-loaded H-ZIF-8/PDA-CD JNPs present higher cellular toxicity compared with mono-drug loaded H-ZIF-8/PDA-CD JNPs and the drug cocktail group. Compared to free JNPs, NIR laser-induced JNPs exhibited better cell-killing properties due to their desirable photothermal conversion features; additionally, cell viabilities for dual-loaded JNPs plus laser were lower compared to other groups, highlighting the effective PTT and chemotherapy synergy. Mice were divided into 12 groups to analyze the in vivo antitumor effects of JNPs: PBS (control group), PBS + laser, free H-ZIF-8/PDA-CD JNPs, free HCPT, free DOX, H-ZIF-8/PDA-CD JNPs+laser, HCPT+H-ZIF-8/PDA-CD JNPs, DOX+H-ZIF-8/PDA-CD JNPs, DOX+HCPT, cocktail, DOX+HCPT+H-ZIF-8/PDA-CD JNPs, DOX+HCPT+H-ZIF-8/PDA-CD JNPs+laser. The temperature of tumor locations was tracked using an infrared camera in both the control and H-ZIF-8/PDA-CD JNPs+laser groups, where laser therapy was conducted for 5 min at 1 W/cm^2^ following delivery for 24 h. This implied that H-ZIF-8/PDA-CD JNPs possess intense PTT efficacy in vivo, since the tumor site in the H-ZIF-8/PDA-CD JNPs + laser group acquired much greater temperature compared to that of the control group. Tumor volumes in the DOX and HCPT co-loaded HZIF-8/PDA-CD JNPs group are much less than those in the solely DOX or HCPT-loaded H-ZIF-8/PDA-CD JNPs, cocktail, along with merely laser and control groups, demonstrating that the co-loaded JNPs plus laser was considerably more effective in suppressing tumor growth. After 24 h of injection, ICPAES measurements showed an uptake of 8.6% ID/g for H-ZIF-8/PDA-CD JNPs in the tumor site, which is much higher than the uptake in other tissues. H-ZIF-8/PDA-CD JNPs were also found in drastically reduced numbers in reticuloendothelial organs such as the liver and spleen. Next, the abundant Zn found in the mice’s urine and feces demonstrated that the HZIF-8/PDA-CD JNPs had been eliminated from the body. Therefore, H-ZIF-8/PDA-CD JNPs can be used as potent antitumor in the treatment of cancer due to their superior biocompatibility and the synergistic effects of combination chemotherapy and PTT [197].

It is also noteworthy to investigate the internalization aspects of the aforementioned study, which examined the dual delivery of 5FU and quercetin in a Janus nanoparticle of PCL/terpolymer and the nanoparticles’ mixed design [159]. The cytotoxicity of dual-loaded Janus nanoparticles was assessed by MTT assay on C6 and OLN-93 cell groups in the study. Analysis of cell viability after 24 h of incubation at 37 and 40 °C with varying concentrations of 5FU, quercetin, 5FU/quercetin, drug-free nanoparticles, and drug-loaded nanoparticles revealed that the drug-free Janus particles have limited toxicity, particularly at higher concentration levels, and do not exhibit a temperature-dependent increase. Neither quercetin, 5FU, nor their combination decreased cell growth at either of the two temperatures tested, implying that neither drug is more toxic than the other. Given the significant increase in solubility and bioavailability of quercetin in the nanoparticles mentioned before in the solubility section, it appears that quercetin-loaded Janus nanoparticles are more toxic than free drug at both temperatures. In addition, the cytotoxicity of Janus nanoparticles loaded with 5-fluorouracil (5FU) was diminished, a phenomenon attributed to interactions between the drug and the terpolymer. The dual-loaded Janus nanoparticles were less toxic than the free mixture of drugs when maintained at 37 °C but much more toxic when heated to 40 °C, especially at higher concentrations of 100 and 500 M, illustrating the promising application of the dual-drug systems in cancer treatment at increased temperatures. Normal cells and the OLN-95 cell line were also tested to determine the inhibitory impact of pharmaceutical combinations in two systems. While both nanoparticles were impactful in inhibiting the reproduction of normal cells, the effect was more pronounced in cancerous cells. Additionally, normal cells were found to be sensitive to the structure of mixed nanoparticles and inhibited in a manner that depended on the nanoparticles’ morphology. Internalization analysis employing optimal microscopy confirmed that mixed nanoparticles were more likely to enter normal cells at 37 °C, while Janus nanoparticles exhibited the opposite behavior at higher temperatures. This was explained by the fact that the interaction between Janus nanoparticles on the surface of cells collapsed, allowing for the entry of individual nanoparticles [159].

**Figure 13:**
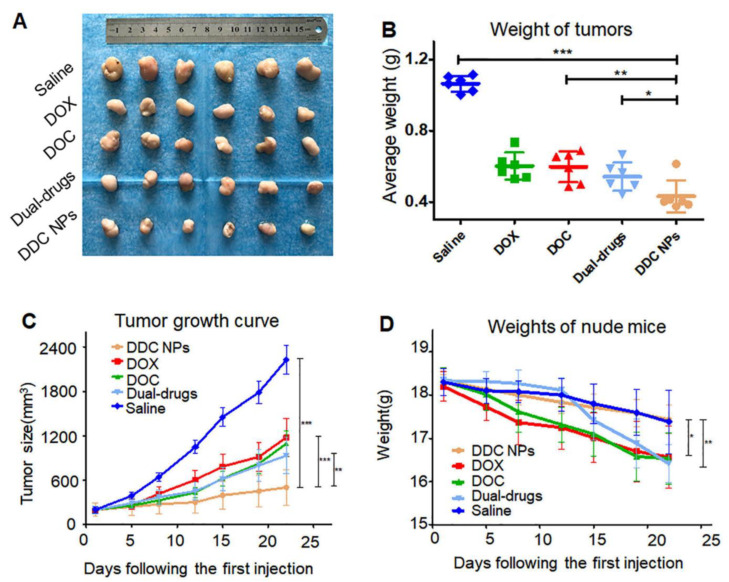
Anti-tumor efficiency of nanoparticles. (**A**) Images of tumors. (**B**) Tumor weight treated by each group. (**C**) Growth patterns of tumors. (**D**) Body weight changes affected by test groups. (For statistical significance, the notation of * represents *p* < 0.05, ** represents *p* < 0.01, and *** represents *p* < 0.001). Reproduced from Li et al. [200], © 2019 Li, Zhan, Chen, Jha and Chen.

As one of the most frequent cancers worldwide [198], prostate cancer has been the focus of several dual-drug treatments, for example, the combination of doxorubicin (DOX) and docetaxel (DOC) [199]. Based on the research of Li et al., DOC was encapsulated in cationic amphipathic starch (CSaSt) via hydrophobic interactions, and nanoparticles of DOX/HA were absorbed on the initial particles to improve tumor internalization at an optimum therapeutic dose. The presence of hyaluronic acid (HA) and prostate-specific membrane antigens (PSMA) ligand–receptor interactions would make nanocarriers containing HA nanoparticles in their structures more capable of targeting and quickly penetrating cancerous tissue. In vitro analysis of the cytotoxicity of DDC NP was performed using three PCA cell lines (PC-3, DU-145, and LNCap), and the CCK-8 assay findings confirmed that the DDC NPs might exert a synergistic influence on both androgen-dependent and androgen-independent PCA cell lines, particularly in PC-3 cells, as confirmed by clone formation assays. Flow cytometry used for the determination of DDC NPs’ effect on PCA cell apoptosis displayed that DDC NPs produced an apoptosis ratio comparable to the combined drug treatment group but significantly greater than that seen with the single drugs. Western blot analysis results of the enhanced pro-apoptotic factor cleaved Caspase in the DDC NP-treated group, along with the much lower ratios of Bcl-2 and Bax in the DDC NP-treated group compared with the single-drug-treated groups, further confirmed that DDC NPs could successfully trigger apoptosis. The DDC NPs were shown to be internalized by a fluorescently labeled, competitively inhibited endocytosis assay. Accordingly, after 30 min, the signals had separated to the extent that the red fluorescence signal (emitted by DOX) had accumulated equally in the cytoplasm and the nucleus, while the green signal (emitted by coumarin-6) had remained in the cytoplasm. By 6 h, almost all DOX had entered the nucleus, whereas coumarin-6 continued to remain in the cytoplasm, demonstrating that DDC NPs had successfully delivered their payloads into the cell through ligand–receptor mediations. Acute toxicity results revealed that DDC NPs attenuated the toxicity of DOC and DOX in vivo, with 20% vs. 80% mortality in mice treated with the combined drug treatment. Tumor suppression experiments employing PC-3 cell xenograft mice showed that while DDC NPs significantly inhibited the development of PCA, none of the single-drug or dual-drug combination groups displayed sufficient tumor suppression in the animal models with a higher average tumor volume than the DDC NPs group (6-fold, 5-fold, and 2-fold volume growth for single drug, dual drug, and DDC NPs, respectively) (Figure 13). Accordingly, the increased accumulation of medicines in tumor tissues and less generic accumulation are potential attributes of DCC NPs for PCA therapy [200].

With the purpose of “multimodal analgesia” investigations and using the double emulsion/solvent evaporation process, resveratrol (Res) [201] and α-Cobrotoxine (αCT) [202] as central pain analgesia medications were encapsulated in an asymmetric Janus design of low molecular weight chitosan/sodium alginate/PLGA, which were controlled to create Janus structures in contrast to core–shell ones. In order to evaluate the oral absorption pathways of αCT/Res-JNP, cellular uptake and trans-monolayer transport investigations were performed. The results indicated that FITC-αCT was absorbed by Caco-2 and HT29/MTX cells only when bound to JNP (FITC-αCT-JNP and FITC-αCT/Res-JNP), but not when free FITC-αCT was present. Additionally, the uptake of cells and the fluorescence intensity of FITC-αCT were not distinguishable between JNP loaded with a single drug and JNP loaded with a double drug, suggesting that the uptake of a single drug was not affected by double drug loading. Moreover, adsorption of JNPs to the Caco2-HT29/MTX-Raji cell models simulating epithelial cell was investigated, where a comparison of free Res and JNP-encapsulated Res revealed no significant difference after 4 h in Caco-2 cells, indicating superior higher osmotic transport of Res due to its hydrophilic nature. The Caco2-HT29/MTX-Raji cell model also displayed higher cumulative transport of free FITC-αCT (almost 4%, 4 h), free Res (nearly 14%, 4 h), and JNP (13–17%, 4 h), which may be due to the combined actions of epithelial cells, the mucus layer, and M cells helping with the enhanced JNP transport. Studies on intestinal absorption and enterocytic uptake further demonstrated that encapsulating medicines in the JNPs would increase their intestinal absorption, as indicated by the higher cumulative uptake of Res and αCT compared with their free-form groups. There is a consistent pattern of enhanced absorption of αCT and Res in the ileum and jejunum and diminished uptake in the duodenum and colon, most likely as a result of JNP’s entrapping abilities. The bio-adhesive properties of chitosan are thought to be responsible for JNP’s membrane-crossing abilities; this property permits drugs to attach to the mucous membrane rather than the intestinal canal, slowing the pace at which the drug is degraded. Fluorescence imaging of intestinal absorption, PK evaluations, and in vivo cell uptake findings indicate that intestinal epithelial cells can transport αCT/Res-JNP to the bloodstream, boosting the dual drugs’ oral bioavailability [203].

**Figure 14:**
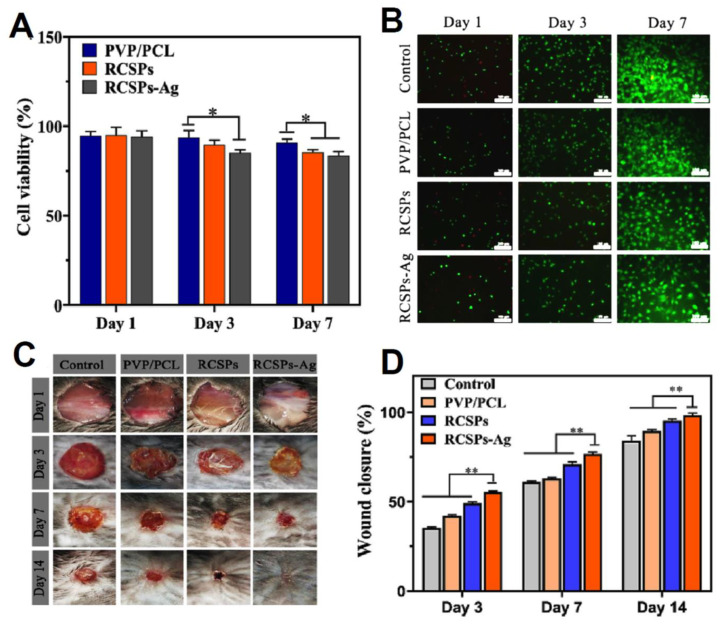
(**A**) Percentage of cell viability of NIH 3T3 cells treated with PVP/PCL, RCSPs, and RCSPs-Ag in different time intervals. (**B**) Confocal laser microscopy images of NIH 3T3 in different test groups. (**C**) Wound healing process images of each group within 2 weeks of treatment with various groups. (**D**) Wound Closure percentages treated with PVP/PCL, RCSPs, RCSPs-Ag nanofibers, and a control group treated with saline gauze (* *p* < 0.05, ** *p* < 0.01). Reproduced from Ji et al. [206], with permission from Elsevier.

Utilizing the encapsulated antibacterial and biocompatible Ag NPs [204], as well as the anti-inflammatory and cell growth-inducing qualities of Rana Chensinensis Skin Peptides (RCSPs) [205], Janus nanofibers were created by uniaxial electrospinning PCL and PVP polymers for use as a skin dressing to promote wound healing. Deionized water contact angles tested the wettability of PVP/PCL, RCSPs, and RCSPs-Ag Janus nanofibers. PVP’s polyamide and vinyl groups make the PVP/PCL nanofiber membrane hydrophilic; while RCSPs and RCSPs-Ag nanofibers were less wettable, the RCSPs-Ag nanofibers exhibited hydrophilic properties, facilitating cell adhesion. Hydrophilic PVP allowed RCSPs-Ag nanofibers to fully absorb water, despite insoluble fiber coverings. The CCK-8 assay on NIH 3T3 cells revealed cell viabilities of 94.68%, 95.12%, and 94.02% for PVP/PCL, RCSPs, and RCSPs-Ag nanofibers, respectively (Figure 14). However, after a week, the cell viability of RCSP and RSPC-Ag nanofibers reduced as a result of drug release into the growth media, revealing the possible adverse effects of Ag on survival rate and biocompatibility. Additionally, *E. coli* and *S. aureus* were used to examine the nanofibers’ antibacterial function, which is a crucial aspect of wound healing. PVP/PCL and RCSP nanofibers exhibited no antibacterial properties; however, Ag-containing nanofibers showed antibacterial activity, albeit at a lower level for *S. aureus* than for *E. coli*, maybe due to surface variations between the two bacteria. Moreover, important healing efficiency of the dual-loaded nanofibers in mice found injury closure rates for the RCSPs-Ag nanofibers, control, PVP/PCL nanofibers, and RCSPs nanofibers to be 98.41%, 86.09%, 89.54%, and 95.4%, respectively, after two weeks (Figure 14). Due to the presence of Ag NPs, which not only had anti-bacterial effects but also improved blood coagulation when combined with RCSP in the Janus structure, the improved performance of RCSP-Ag nanofibers related to the other groups can be explained. Intriguingly, the breakdown of PVP upon contact with bodily fluids caused the nanofibers to change from a Janus structure to a three-dimensional grooved structure, resulting in increased porosity and a higher surface-to-volume ratio, which facilitates cell adhesion. C57 mice were divided into four groups and subjected to histopathology on days 3, 7, and 14. Day 3 showed extensive inflammatory cells in the control and other groups but less inflammation in the RCSPs-Ag group. The RCSPs-Ag group showed the highest levels of vascular healing and fibroblast proliferation by day 7, and complete re-epithelialization and new hair follicles by day 14. In conclusion, due to their wettability, skin-related mechanical properties, antibacterial efficacy, and biocompatibility, the Janus nanofibers exhibited extraordinary potential for use in wound healing applications [206].

Patchy nanoparticles, as an interesting structure in anisotropic materials, also can be loaded with multiple drugs in their specific patches, according to a study by Wong et al. in which triblock terpolymers of poly (glycosyloxyethyl methacrylate)-*block*-poly (benzyl acrylate)-*block*-poly(4-vinylpyridine) PGlcEMA_94_-*b*-PBzA_277_-*b*-P4VP_370_ were synthesized using RAFT polymerization and then self-assembled into the patchy structure with P4VP core decorated with multiple patches of PBzA containing PGlcEMA corona. In this structure, hydrophilic DOX was loaded in PBzA as the more non-polar section of the polymers chain, while Cyanine5 (Cy5) fluorescent dye as the model drug was able to bond to the more hydrophilic P4VP through forming hydrogen bonds. Additionally, due to the loading of fluorophores into separate compartments of nanoparticles, fluorescence resonance energy transfer (FRET) occurred at the sites where the PBzA patches connect to the P4VP core, enabling the monitoring of real-time release in cells. As Cy5 is already known to be harmless and non-toxic, the toxicity analysis of Cy5-loaded patchy particles was omitted from the research. However, cell survival experiments employing the MCF-7 breast cancer cell line revealed that empty patchy particles were non-cytotoxic; however, DOX-loaded patchy particles were toxic with an IC_50_ value of 15.3 g/mL, equal to 0.69 M of DOX concentration. The successful internalization of the drug-loaded particles in the cytosolic sites of MCF-7 cells was also approved by confocal microscopy. Monitoring real-time release from the patchy particles was reported after 120, 150, and 180 min of MCF-7 cells incubation with co-loaded patchy nanoparticles, anticipating the decreased FRET signal after slow release as a result of the separation of the fluorophores. Some of the patchy particles co-loaded with DOX and Cy5 survived internalization, as evidenced by the presence of the FRET signal at all three time points. Accordingly, the numerical analysis of the FRET efficiency variations over time showed a linear decrease in FRET efficiency, indicating that the co-loaded DOX and Cy5 were being released into the cells progressively from the distinct compartments of the particles. In 120 min of incubation, the cell line was healthy, and by 180 min, bubbles had developed on the cell membranes, in agreement with the FRET efficiency results. This was attributed to the loaded DOX that released slowly, leading to a steady rise in DOX concentration within the cells and, ultimately, the apoptosis phenomenon [207].

Notably, all the reviewed research data of anisotropic structures are listed in Table 2, providing the summary of information at a glance (Table 2).

## 4. Discussion

Nanoparticles, given their numerous beneficial features, hold great potential for the targeted administration of drugs in the treatment of a wide variety of ailments from Alzheimer’s disease and Parkinson’s disease to different types of cancer [208]. The unique and essential features of nanoparticles, along with their ability to absorb and transport other chemicals, make them promising for use in medicine. Particles may absorb and store a wide variety of chemicals, including drugs, probe agents, genes, and proteins, on their surface, in their matrix, or in their core. Co-delivery of multiple cargos using nanoparticles, however, provides additional advantages, namely synergic effect where two anticancer drugs can operate on distinct routes at the same time, increasing cancer cell death and preventing MDR, targeted tumor apoptosis and reduction in side effects [209]. Some of the parameters that may be effective in choosing the most appropriate carrier type used in drug delivery include size, surface design, and simplicity of the synthesis process. [210].

Yet, additional factors should be taken into account in the structures utilized for dual-drug delivery systems, namely the interactions between the nanocarrier and cargo. Time and the mechanism by which the cargo is released are both influenced by these interactions. By choosing the appropriate interactions depending on the target tissue and the combination of pharmaceuticals, the ideal release mechanisms and time for the drugs may be obtained for optimum efficacy. For instance, in [108], CUR was connected to the micelle’s backbone by S-S bonds. The resulting ALN-oHA-S-S-CUR self-assembled into micelles with a CUR core where the medicines could be released via disulfide bond cleavage in the reducing environment of the cells. Nevertheless, the mentioned desired situation is susceptible to another critical aspect which is the nanoparticle architecture to allow for separate locations for drugs loading without possible undesirable interactions with each other. However, this depends on the type of loading, which might be totally segregated in distinct locations as in most anisotropic nanoparticles, or in cocktail/mixed forms, loaded in the same location, as in [119], where DOX and TPL were stored in the same area. The ideal structure may eventually result in different release methods and timings, such as sequential release mostly in anisotropic structures [31], or simultaneous release in both uniform and anisotropic NPs. To facilitate evaluation and selection, it is important to have precise data on the target tissues and pharmaceuticals, as well as the required qualities in terms of release type and performance of nanoparticles.

For example, the release sequence and quantity of doxorubicin (DOX) and paclitaxel (PTX) from Au-MSN Janus NPs with a β-CD decoration on the gold layer, reviewed in [153] depended on pH variations and NIR laser presence. The dual-loaded JNPs released more DOX in acidic conditions and PTX up to 62.74%. At pH 5, where DOX release was around 73% regardless of NIR laser induction, PTX release improves by 5% due to cleavage of the Au-S bond, and NIR heat also promotes PTX release from the β-CD by cleaving the PTX-β-CD bond. In [166], another anisotropic SiO_2_-based NPs formulation was tested with the same drugs. Drugs (PTX and DOX) stored in the PMO area may be released at temperatures over the PCM melting point and according to the cis-to-trans and trans-to-cis transitions of UCNP compounds under UV and visible irradiation. The dual-drug-loaded UCNP@SiO_2_@mSiO_2_-Azo&PMO-PCM Janus nanocomposites against cancer cells resulted in enhanced cytotoxicity when subjected to heat treatment (39 °C). The initial switch causes the PCM to melt, which allows the paclitaxel molecules to escape via the mesopores of PMO. Apparently, it was also possible to boost cytotoxicity by releasing paclitaxel and DOX molecules simultaneously through the application of heat and NIR light. The cell viability of this structure against HeLa cells was less than 30% at simultaneous release and nearly 70% with only heat (first switch on, PTX release), while in the case of the former example, Au-SiO_2_ JNPs, the improved release efficacy of pharmaceuticals on cell death was demonstrated by in vitro cell viability data displaying less than 15% cell viability of SMMC-7721 cells following incubation with dual-loaded JNPs with NIR laser irradiation (808 nm, 2.0 W/cm^2^, 5 min).

PTX and DOX were evaluated again for their release and drug–drug interaction effects on release patterns, however, this time loaded in a multicompartment hydrogel (MCH) [179]. Despite being released from the same carrier, the diffusion rates of the two medications did not impact each other, demonstrating the absence of drug–drug interferences in the former comparison. Especially for the release of DOX, which has a diffusion release mechanism due to the slow degradation of the fluorocarbon portion. By using this formulation, any medicine within a certain concentration range might be delivered locally and continuously. Notably, the drug–MCH cocktail significantly increased the cytotoxicity against MCF-7 cells in vitro, with less than 10% cell viability after 7 days (Table 2). Considering the three samples with the same combination of drugs and their varied results demonstrated that only by changing the shape and composition can the ultimate performance in release be determined.

By comparing structures of pharmaceuticals or drug compounds that are nearly comparable, it is evident that a general comprehension of performance may be obtained. Nevertheless, additional aspects such as the potential of structure for mass production, the target tissue, and the availability of external stimulation elements such as UV or NIR laser or magnetic power should also be considered. For instance, the treatment of hepatocellular carcinoma (HCC) using dual delivery systems was the subject of two different studies. The types of nanoparticles and drugs, however, varied in the studies, where in [132], DOX and curcumin were loaded in the uniform nanoparticles of magnetic nanoparticles functionalized with β-CD-MA-PNIPAM copolymer. MTT assay using cervical cancer cell line (HeLa) results demonstrated that, at concentrations of 10 and 50 g/mL, drug-loaded nanoconjugates significantly reduced cell viability (maximum at 50%), especially when exposed to a magnet (~below 50% cell viability). Apparently, the capacity of NPs to rapidly attain the crucial temperature for killing cancer cells upon induction of an external magnetic field contributed to hyperthermia therapy. Moreover, high cytotoxicity of curcumin was observed using the structure owing to the capability of nanoconjugates to bring high doses of medicines into the cell through endocytosis. Due to the increased absorption of the nanoconjugates by cells caused by the folate ligand functionalization, treatments with curcumin and DOX resulted in considerable cell death and a notable reaction in the form of more cells expressing PI-positive staining. However, in [196], DOX and berberine (BER) were loaded onto anisotropic Janus HA-modified magnetic mesoporous silica nanoparticles. Dual-loaded HA-MSN@DB with DOX and BER suppressed HepG2 and HL-7702 cells in a dose-dependent manner. This combination also showed the highest apoptotic efficiency (48.10%) among other systems, significantly reduced tumor growth, and increased mouse survival. Both studies took different paths in the treatment of the same ailment, using a varied combination of medicines and nanocarrier designs. However, the latter nanoparticles were created with the hope of taking advantage from the theory that such a carrier with rod-shaped architecture, in contrast to conventional spherical core–shell structures, would result in increased intracellular internalization and tumor accumulation and BER capability of reversing doxorubicin (DOX)-induced HCC regeneration in vitro and in vivo.

Importantly, both examples demonstrate the impact of ligands and their contribution to enhanced internalization and targetability properties of dual-drug delivery systems. In β-CD-MA-PNIPAM, the targetability of the nanocarriers was facilitated by the high affinity of folic acid ligands for overexpressed folate receptors on tumor cells, where the nanoconjugates may penetrate the plasma membrane of HeLa cells and end up inside the cytoplasm. Nevertheless, in HA-MSN@DB NPs, the targetability was aided by the nanoparticles’ specific affinity for CD44 receptors. It is also important to note the effect of external magnetic or NIR/UV laser inductions on the improvement of internalization and release rates. As an example, the release rates of DOX and GCT loaded in PNIPAM-b-PNAM-b-PNBOC improved from less than 20% of GCT and 10% of DOX without UV to 98% of GCT and 80% of DOX in the presence of UV irradiation [180]. The same beneficial impact can be observed in many reviewed studies, specifically in the case of more apoptotic characteristics, such as the case of BP@PDA-PEOz loaded with DOX and BTZ, where with 808 nm NIR laser light, over 80% of MCF-7 cells were killed, demonstrating the photothermal application of the design with the help of NIR irradiations [141]. Although these characteristics would be relevant to all types of delivery systems, whether they contain one or more than two medications, incorporating them into a dual delivery system might enhance the synergic cytotoxic impact if the release rates or targetability are enhanced by other means, such as ligand functionalization.

In the ideal instance, the set of cargoes in the structure of the same type is transported towards the same target, allowing for a thorough analysis of the functional difference between multiple drug delivery in uniform or anisotropic structures. This will allow for a comprehensive review of how the variability in drug loading space affects release, synergistic characteristics, and internalization. The paper by Khoee et al. referenced before provides this analysis [159]. (PCL) and (N-isopropylacrylamide-co-acrylamide-co-allylamine) terpolymers were applied to the surface of GO, resulting in two distinct structures: a Janus structure, formed when the polymers were placed in an asymmetrical fashion, and a mixed structure, formed when the polymers were not positioned in distinct sections. These nanoparticles were employed to transfer 5FU and QCT, and their efficacy was assessed by comparing their in vitro release patterns and cytotoxicity to cells at 37 and 40 °C. Evidently, more than 80% and nearly 80% of 5FU, and nearly 40% and 50% of QCT were released from dual-loaded uniform and Janus nanoparticles at 40 °C, respectively. Janus nanoparticles, which contain two pharmaceuticals, significantly reduced the toxicity to C6 cells as measured by the MTT assay at 37 °C (roughly 40%) in comparison to the free combination drugs, while they are more toxic than free drugs under the same conditions (nearly 20% cell viability at 65 µg/mL concentration) at 40 °C, highlighting the beneficial impact of multiple medications on the treatment of cancer cells using the Janus structure at higher temperatures. The dual-loaded uniform nanoparticles however showed less cell viability than Janus counterparts, (20% at 37 °C, and less than 20% at 40 °C), and more cytotoxic effect. Furthermore, the degree of inhibition was greater for uniform nanoparticles, and this was also a function of the morphology of the nanoparticles; in other words, the nanoparticles themselves determined the effectiveness and internalization of the dual drug-loaded nanoparticles. Janus nanoparticles created big aggregates on the cell surface at low temperatures, which may account for the higher number of internalized uniform NPs than Janus NPs. Interestingly, the Janus nanoparticles’ interparticle interactions were destroyed at increased temperature, allowing the individual nanoparticles to have a greater chance of being internalized into tumor cells. All in all, these findings indicated that the different morphologies of the two nanoparticle types are responsible for the differential effects on cancer cells, controlled by variations in temperature.

Our findings indicate that the optimal structure for simultaneous drug administration is very context-dependent, depending not only on the substances being administered but also on the purpose for their distribution, their target tissue, the ease of their carriers’ synthesis, and the nature of their interactions with one another. When other factors, such as the manufactured particle composition, drug combination, and target tissue, are similar, the desired surface morphology can be chosen to be uniform or non-uniform. Nevertheless, in addition to the significant properties of the desired particles for drug delivery in single-cargo structures, such as functionalization with surface ligands or proteins that increase internalization as well as external powers including UV and NIR irradiation and release control via pH variations, considering the suitable loading location for numerous cargos at the same time would have a significant and unique influence on the synthetic performance of pharmaceuticals.

## 5. Challenges and Outlook

Significant difficulties arise while analyzing the uniform and anisotropic drug delivery of multiple drugs simultaneously, a topic that has been widely overlooked in the research. The detailed comparison of studies requires the utilization of a wide range of nanoparticles with varying compositions, surface structures, morphologies, and cargos so that different results may be derived from comparing the efficiency of structures and their medicinal content according to their similarities and differences. As noted in the introductory section of this article, the shortage of research on various structures of this kind is one of the most significant obstacles to analyzing the outcomes of dual-drug delivery, specifically using anisotropic structures, where the majority of publications are focused on Janus structures while the number of such studies employing heterogeneous multicompartment and patchy structures is substantially smaller. Consequentially, a limited number of pharmaceuticals or drug compounds have been studied in these forms, which makes comprehensive evaluation of the structures much more challenging. This may be a consequence of the difficulties of synthesis methods and related techniques for synthesizing two or more sections with distinct properties within a single particle [211]. Examples include patchy particles, where it is still challenging to attain the precise arrangement of patches of varying kinds on the surface of a colloidal particle, which is essential for the creation of multi-shell capsids [212]. In addition, there is a greater range of Janus nanomotors, including Janus micro/nanomotors that have been extensively studied in drug delivery systems [213,214]. Although these particles make use of the capabilities of hybrid and anisotropic structures as well as unique qualities such as spontaneous movement with directed trajectories and controlled release employing optical, magnetic, and electric forces, research on their uptake and transfer of two or more drugs is neglected.

Nevertheless, it is worth noting that synthesis techniques for the uniform material also present obstacles, such as scaling-up difficulties with widely used liposome nanoparticles’ synthesis using thin-layer hydration or reverse phase evaporation methods and the difficulty of removing byproducts or unwanted material from the polymeric nanoparticle reaction medium [209].

Moreover, the majority of dual-drug delivery studies are based on a single structure, and the combination of medications or intended cargoes is only studied in a single structure. While analyzing the efficacy of medication combinations, the synergistic effects, internalization, and release of pharmaceuticals in two structures may offer a better view of the performance differences between structures with the same chemical composition. As another challenge, nanoparticles are studied less often to improve the solubility or dispersibility of pharmaceuticals in systems containing more than two medications. Despite the relevance of solubility and dispersibility of the medications in dual-drug delivery to their improved bioavailability and performance, studies on these systems do not provide detailed data on the enhancement of drug solubility along with relevant analyses in these systems. The effectiveness of nanoparticles with uniform or anisotropic structures in improving the solubility of pharmaceuticals with variable hydrophilic or hydrophobic qualities is limited to mere generalizations, whereas a detailed examination of these features in diverse media with varying pHs would be of considerable assistance in the clinical and commercial use of these dual-drug delivery systems.

Notwithstanding existing challenges, significant progress has been achieved in the synthesis of anisotropic structures using more precise and more ordinary techniques, contributing to the creation of a variety of patchy, Janus, and multicompartment structures that will be the subject of further study. Electron beam deposition, for instance, allows for the regulated creation of particles with varying patch geometries; hence, one path for future study is to explore the possibilities of resultant materials in the delivery of numerous cargos. Physical vapor deposition (PVD), which is extensively compatible with inorganic and organic particles of various sizes and shapes [215], permits the straightforward deposition of asymmetric compartments on nanoparticles, where the modification in the angle of deposition (GLAD method) [216], generates multiple sections with the appropriate forms. Each of these developments in the creation of asymmetrically surfaced nanoparticles provides a new avenue for investigation into improved co-delivery methods, eventually contributing to possible clinical tests determining the effectiveness of co-delivery designs in the treatment of diseases, namely cancer.

## 6. Conclusions

This article provides a summary of the research performed on the topic of concurrent drug administration in a nanoparticle with a unique surface structure. The interaction between the cargo and substrate, how it is released, how it binds to the cell, etc., were studied in two uniform and anisotropic structures to achieve a greater understanding of the behavior of these structures during the simultaneous movement of two therapeutic medications. This level of specificity can aid in the selection of the most effective substrate or medication for a certain application, whether it be for simultaneous or sequential loading and release, evaluation of drug performance in various structures, or the destruction of malignant cells.

## Figures and Tables

**Table 1:** An instant overview of the mentioned uniform dual delivery systems.

Nanocarrier	Cargo	Outcomes and Details	Ref.
PLGA NPs	diclofenac sodium (DS) and dexamethasone (DX)	Higher solubility and cell uptake for both drugs. Encapsulation efficiencies were (EE%) 67.7% for DX and 54.2% for DS (drug/polymer 1:10 ratio). Release rates were 80% for DS and 60% for DX. Cytotoxicity data revealed nearly 9% reduction in cell viability compared to the control group with 69.4% viability.	[53]
GO functionalized with poly(epichlorohydrin)-graft-hyperbranched polyglycerol (PCH-g-HPG) NPs	doxorubicin (DOX) and curcumin (CUR)	high dispersibility and biocompatibility particularly for CUR, drug loading efficiency (DLE) was highest at 85% for DOX, and at 80% for CUR. The release rate was nearly 90% for dox and 45% for CUR at pH 5. cytotoxicity on MCF-7 displayed nearly 20% cell viability for NPs loaded with both drugs compared to that of the 70% and 50% loaded with only CUR or DOX, respectively.	[56]
mPEG-b-P(Glu-co-Phe)	doxorubicin (DOX) and curcumin (CUR)	improved encapsulation and solubility of the CUR. Drug loading was calculated to be 9.7% for DOX and 8.1% for CUR, with DOX: CUR 1: 1.23 (molar ratio). DOX release after 60h at pH 5, 6.8, and 7.4 was reported 60%, 30%, 20%, respectively. Less cell viability (in most of the samples) and higher apoptotic rates in NPs with both drugs was observed compared to single-drug samples.	[57]
methoxy-poly (ethylene glycol)-block-poly lactic acid (mPEG-b-PLA)	curcumin (CUR) and bortezomib (BTZ)	enhanced solubility and cytotoxicity for both hydrophobic drugs specially CUR, 20–26% drug loading efficiency regarding the copolymer composition, higher cytotoxic effect of curcumin–BTZ complex loaded NPs for the MCF-7 cell line compared to the free curcumin–BTZ were observed. The 8k NPs were significantly more toxic compared to free complexes, and NPs with the shortest lactide chains were more cytotoxic.	[61]
mesoporous silica nanoparticle (MSNs)	doxorubicin and camptothecin (CPT)	sequential release, selective internalization. 25% DOX and 6.3 ± 3% CPT-PEG were loaded. No release at pH 7 was observed for both drugs while the release was 25% and 15% at pH 5.5 for DOX and CPT-PEG, respectively. HepG2 cells viability was 17% when using CPT-PEG@MSN-DOX compared to single-drug MSN-DOX cell viability at 47%. Selectivity of cytotoxic effect towards HepG2 cells was observed when NPs were decoreated with glycyrrhetinic acid (GA).	[74]
mesoporous silica nanoparticle (MSNs)	paclitaxel (PTX) and MDR reversal agent tetrandrine (TET)	pH-responsive release property, markedly increased intracellular accumulation of NPs. The drug loading efficiency was 7.23% for PTX and 1.21% for TET in NPs loaded with both drugs. pH-responsive release where release rates were 7.5% for PTX and 16.5% for TET after 72 h at pH 7, while release percentages of PTX and TET were 53.9% and 67.9%, respectively. More cell deaths and proliferation inhibition in the combination of two drugs using NPs than free drugs indicating better endocytosis and uptake of the drugs using MSNs.	[81]
magnetic mesoporous silica nanoparticles	doxorubicin (DOX) and hydrophobic drugs paclitaxel (PTX) and rapamycin (RAPA)	enhanced internalization, tumor cell apoptosis, and growth inhibition as compared to single-drug loaded MMSNs. The slower release was observed for DOX from DOX-PTXMMSNs (23%) and DOX-RAPA-MMSNs (19%) compared to the single drug (25%). Apoptotic ratios in cells were 30.72% for DOX-PTX-MMSNs and 47.30% for DOX-RAPA-MMSNs greater than the apoptotic ratios induced by each single-drug-loaded MMSNs.	[87]
thermally hydrocarbon sized-Psi (THCPSi) and thermally oxidized-PSi (TOPSi).	indomethacin (IMC), and peptide tyrosine tyrosine 3-36 (PYY3-36)	co-loading of therapeutics enhanced the loading capacity of the PSi NPs, accelerating the release rate and permeation. TOPSi-IMC-PYY showed a loading degree of 15.0 ± 0.1% for PYY3-36 and 13.7 ± 0.4% for IMC, and THCPSi-IMC-PYY with 18.4 ± 0.8% for PYY3-36 and 16.8 ± 0.8% for IMC. release rates of IMC and PYY3-36 from co-loaded PSi NPs were faster than single-drug-loaded ones at pH 7.4 and 5.5. Improvements in the cell viability rates were observed for the PSi NPs loaded with PYY3-36 compared to the plain PSi NPs.	[50]
FMSN@PDA@GO	ibuprofen (ibu) and acetaminophen (acet)	sequential release with individual kinetics for each drug. Loading amounts were 0.4 wt. % and 6.1 wt. % for MSNs-Ibu and FMSNs-Ibu, respectively. The cumulative release fraction was 90% at 36 h for Ibu and 15 h for Acet, respectively. Effective blocking layer of PDA @GO coating prevented rapid transmission of acetaminophen.	[92]
PEG@MSN nanorods	camptothecin and survivin shRNA-expressing plasmid (iSur-DNA)	higher targetability, uptake, and cytotoxicity owing to AS1411 aptamer. In total, 32% of encapsulation efficiency for CPT was reported. In total, 65% and 16% CPT was released from PEG@MSNR-CPT in citrate buffer (pH 5.4) and PBS (pH 7.4), respectively. The cytotoxicity of Apt-PEG@MSNR-CPT/Sur (20% cell viability) was significantly more than non-targeted NPs in the C26 cell line.	[98]
MSN modified with N-(propyl)aniline and β-CD cap	5-fluorouracil (5FU) and naproxen (NAP)	pH-responsive release in acidic condition controlled by CD, and more toxicity of drugs loaded in the nanoparticle. The total amount of drugs loaded in the SBA-15_N-ANI_5-FUN_ β-CD sample was 20 mg of 5FU and 62 mg of NAP per 1 g of solid. Less and slower release rates was detected in neutral pH due to the closed gatekeepers. The viability of U87 MG cells at pH = 6.3 was remarkedly reduced by the administration of SBA-15_N-ANI_5-FUN_β-CD (5FU + NAP 1:1)	[100]
MSN-hyd-PEG-hyd	doxorubicin (DOX) and camptothecin (CPT)	proper cytotoxicity of the dual delivery systems induced by pH-triggered release. Loading rates were 3.1% for CPT and 25% for DOX. Negligible release was detected at higher pH and physiological pH while nearly 10% of DOX and 5% of CPT were released in pH 5.5.	[103]
poly alendronate-hyaluronan-S-S-curcumin copolymer (ALN-oHA-S-S-CUR)	curcumin (CUR) and alendronate (ALN)	drug release under the reducing environments, high uptake and cytotoxicity of the active targeting redox-sensitive micelles on MDA-MB-231 cells owning CD44 receptors ALN-oHA-S-S-CUR micelles showed 52.58 ± 8.1 encapsulation efficiencies of CUR. Nearly 70% release from ALN-oHA-S-S-CUR micelles via 10mM GSH. The 40 µg/mL cur-loaded ALN-oHA-S-S-CUR micelles displayed the lowest cell viability after 48 h in MCF-7 cells and MDA-MB-231 cells	[108]
lipid–polymer hybrid nanoparticles (LPNPs)	triptolide (TPL) and doxorubicin (DOX)	Encapsulation efficiencies were 75.5% for DOX and 58.3% for TPL in DOX/TPL-LPNPs. Roughly 78% of the DOX and 70% of TPL were released in the presence of 10 mM DTT. DOX/TPL-1/0.2-LPNPs demonstrated higher cytotoxicity than other samples with 20% cell viability and synergistic effect (CI < 1) at all concentrations.	[119]
protein–lipid nanocomplexes (DTPLNs) (Apoferritin (AFr)/cationic solid lipid nanoparticles (cSLN) )	doxorubicin (DOX) and mitoxantrone (MTO)	At pH 6.8 DTPLNs released 75.99% of MTO and 94.52% of DOX. DTPLNs and TPNs displayed more growth inhibition compared to the free combination drugs with growth inhibition values of 31 ± 3.46, and 17 ± 3.42 (%) for MCF-7 cells, along with the greater cytotoxicity of the DTPLNs than other samples in folate receptor-positive cells due to the higher cellular uptake via FRs mediated endocytosis in PC-3 and MCF-7 lines.	[125]
liposomes (FA-DOX/siRNA-L)	doxorubicin (DOX) and Bmi1 siRNA	Significantly higher inhibitory and cytotoxicity efficacy than sole delivery with the help of targeting ligand. DOX encapsulation in FA-DOX/siRNA-L was 89.3% where the siRNA were fully encapsulated in the liposomes. FA-DOX/siRNA-L displayed improved cytotoxic features, where 90.5% of KB cells, 82% of HeLa, and 68% of Hep3B were killed, and enhanced tumor size reduction.	[131]
Nanocarriers based on β-Cyclodextrin modified with Maleic anhydride and NIPAM	doxorubicin (DOX) and curcumin (CUR)	nanoconjugates enhanced the endocytosis of poorly bioavailable drug curcumin, increased internalization using magnetic NPs. The drug entrapment efficiency of the nanoconjugates was 88% where the drug-loading content of curcumin and DOX was 45 wt% and 32 wt%, respectively. DOX release was Almost 60% at pH 5 and 37 °C vs. more than 80% at pH 5 and 40 °C, while curcumin release was less than 20% at pH 5 and 40 °C vs. 80% at pH 7.4 and 40 °C. Drug-loaded NPs displayed noticeable reduction in cell viability specifically in the presence of magnet, due to the enhanced uptake into the cell via endocytosis.	[132]
poly(AMA-co-IMMA)-b-poly(OEGMA) (PAIPO) copolymers	paclitaxel (PTX) and cis-platinum (Pt (II))	enhanced cellular uptake and apoptosis owing to the charge-conversion of micelles surface. The drug loading content (μmol/mg prodrug) in ^DA^M (PTX1/Pt) was 0.101 for PTX and 0.112 for Pt (II). Approximately 82% of PTX and 88% of Pt (II)were released after 72 h in NaASc 5 mM + GSH 10 mM at pH 5.0. Cell-viability data of the HeLa and Skov-3 cell lines demonstrated the highest synergic effect in ^DA^M(PTXn/Pt), exhibiting the lowest IC_50_ = 0.37–0.79 for HeLa, and 0.41–0.78 for Skov-3.	[140]
Black phosphorus nanosheets (BP @PDA-PEOz)	doxorubicin (DOX) and bortezomib (BTZ)	enhanced the cellular absorption of DOX and enhanced circulation time, possibly due to the charge reversal of PEOz.Higher release rates for both DOX and BTZ at 45% and 97% at pH 5 in the presence of NIR laser. Co-administration of DTX + BTZ being more cytotoxic than DOX or BTZ alone and DOX-loaded BP@PDAPEOz-BTZ with 808 nm laser irradiation (1.0 W cm^−2^) had the lowest rate of survival after 48 h.	[141]
PEGylated bilirubin nanoparticles (BRNPs)	dimer-7-ethyl-10 hydroxycamptothecin (d-SN38) and dimer-lonidamine (d-LND)	Encapsulation efficiencies of 95.33% and 92.34% for d-SN38 and d-LND were observed, respectively. The relative cumulative release of d-LND was 96.27% vs. 40.32% (100 × 103 M H2O2 vs. PBS), while that for d-SN38 was 67.39% vs. 25.95% (0.01 M H_2_O_2_ vs. PBS) indicating ROS-responsive release type. SL@BRNPs/iRGD groups cells apoptosis was at 54.2% (1.12-fold greater than SL@BRNPs), demonstrating the superior absorption of nanoparticles by iRGD and the subsequent increase in cell death.	[136]
Aptamer-Chitosan-AuNPs	5-fluorouracil (5FU) and doxorubicin (DOX)	The optimum DEE(%) for 5FU was 74.59% and at the weight ratio of 0.125:1 (Dox: CS-Au-5FU NPs, the DEE of DOX was 83.67%. Nearly 90% of 5FU and 55% of DOX were released at pH 5.4 from Apt-Dox-CS-Au-5FU NPs after 120h. Higher cellular uptake in aptamer-functionalized NPs, in addition to enhanced proliferation inhibition induced by Apt-functionalized DOX-CS-Au-5FU NPs via less necrosis (7.03%) and a higher percentage of apoptotic cells (23.11%) compared to free drug and other groups.	[144]
chitosan/dextran sulfate/chitosan (CS/DEX/CS) NPs	5-fluorouracil (5FU) and doxorubicin (DOX)	efficient internalization and uptake of PTX and 5FU in HepG2 cells, enhanced inhibition of cancer cells. the encapsulation efficiencies of PTX and 5FU in CS/DEX/CS NPs were 66.3% and 75.2%. Both medicines exhibited pH-sensitive release where at pH 5.67, 6.58, and 7.4, 5Fu release rates were 99.41%, 96.20%, and 87.00%, respectively, whereas PTX release was 51.90%, 40.00%, and 32.09%. the greatest cell inhibitory activity for CS-PTX/DEX/CS-5Fu and higher apoptosis rate of 39.55% for the dual drug-loaded nanoparticles in comparison with single-drug groups against HepG2 cells, indicating the synergic effect of drugs.	[145]
Exosomes	5-fluorouracil (5FU) and miR-21i inhibitor	High cell uptake owing to the exosome fused with HER2 proteins, efficient synergic effect in cell killing compared to single drug. Exosomes’ loading capacity (LC) for 5-FU and miR-21i was around 3.1% and 0.5%, respectively. THLG-EXO penetrated Her2-mcherry-SGC-7901 cells more efficiently than SGC-7901 WT, and a significant reduction in tumor volume was seen in the group treated with the THLG-EXO/5FU/miR-21i compared to the THLG-EXO and THLG-EXO/5FU groups.	[148]
FA-R8-functionalized liposome	astragaloside IV (ASIV) and doxorubicin (DOX)	FA-R8-LPs overcame DOX resistance, exhibited enhanced cytotoxicity. In total, 98.57% and 98.49% of EE% were obtained before freeze-drying. improved internalization and targetability of the final FA-R8-LPs-DOX/AS formulation indicating the optimal function of FA and R8). Tumor volume also was smallest after two weeks of treatment with FA-R8-LPs-DOX/AS.	[150]

**Table 2:** Anisotropic structure dual delivery data in a glance.

Nanocarrier	Cargo	Outcomes and Details	Ref.
Janus Au-MSN NPs with preferential functionalized SH-β-CD on the surface of Au area	paclitaxel (PTX) and doxorubicin (DOX)	The loading efficiency of DOX and PTX on the PTX-AuMSN-DOX JNPs was approximately 58.05% and 95.23%, respectively. DOX was released at 22.31% at pH 7.4 and 73.48% at pH 5.0, while PTX showed cumulative releases of 67.55 and 62.75% in conditions with and without NIR laser, respectively. the dual-drug-loaded system presents high inhibition of tumor, and cytotoxicity with the CI value of 0.81 ± 0.01 indicating the synergistic effect of Janus nanoparticles.	[153]
oleic acid-NaYF4:Yb,Er/polydopamine Au nanoflower Janus NPs	hydroxycamptothecin (HCPT) and doxorubicin (DOX)	The loading efficiency (LE%) of the OA-UCNPs/PDA-AuF JNPs was 88% for DOX and 45% for HCPT. Drug release rates from drug-loaded OA-UCNPs/PDA-AuF JNPs within 104 h were 24% for HCPT and 26.5% for DOX at pH 7.4 vs. 87.1% for DOX and 60.5% for HCPT at pH 5.3. The HCPT/DOX-loaded OA-UCNPs/PDA-AuF JNPs (HCPT: DOX (1:1)) showed the lowest CI values (0.3805), best synergistic effect, and 52.0% apoptotic HepG2 cells using HCPT/DOX-loaded OA-UCNPs/PDA-AuF JNPs, higher than single-drug samples.	[154]
PLGA Janus NPs	paclitaxel (PTX) and doxorubicin hydrocholoride (DOX. HCl)	one-step process of Janus synthesis using a fluidic nanoprecipitation system (FNPS). PTX and DOX in Janus particles each showed an encapsulation efficiency of 80% and 15%. The cumulative drug release was nearly 90% for PTX and more than 70% for DOX from Janus NPs after 120h.	[157]
PLGA/PCL Janus particles	curcumin (CUR) and quercetin (QCT) and acetaminophen (APAP)	The encapsulation efficiency (EE%) in Janus NPs was 93.11% for CUR and 92.03% for QCT, while it was 93.38% for CUR and 86.9% for QCT in PCL. The EE% of the APAP and NPX was reported as 54.9% and 93.98% in O/W with ethyl acetate, 21.04% and 91.88% in O/W with DCM + methanol, and 68.29% and 85.49% in W/O/W. Double emulsion method resulted in high encapsulation efficiency, compartmentalization, drug release control.	[158]
Janus and mixed structures of GO-based PCL/terpolymer NPs	quercetin (QCT) and 5-fluorouracil (5FU)	Encapsulation efficiency (EE%) of QCT and 5FU was 41.91% and 53.25% in Janus NPs, respectively, vs. 38.03% for QCT and 71.77% for 5FU in mixed NPs. More than 80% and nearly 80% of 5FU, and nearly 40% and 50% of QCT were released from m-(PCL-NGO-Terpolymer) and J-(PCL-NGO-Terpolymer) at 40 °C, respectively. The dual-loaded NPs were less toxic than the free mix of drugs when maintained at 37 °C but much more toxic when heated to 40 °C. The inhibitory effect of dual-drug-loaded nanoparticles on normal cells was dependent on nanoparticle morphology, with mixed nanoparticles showing the greatest degree of inhibition.	[159]
polystyrene/Fe_3_O_4_ @SiO_2_ Janus nanocomposites (SJNCs)	Fe_3_O_4_ and doxorubicin (DOX)	The loading efficiency of ≈2% (*w*/*w*) was calculated for DOX, with release rates of 25.1% (*w*/*w*) at pH 7.4, 47.1% (*w*/*w*) at pH 6.0, and 82.6% (*w*/*w*) at pH 5.0. The apparent cytotoxicity of DOX coupled to non-targeted Janus particles was 5-fold reduced when compared to free DOX, while FA-functionalized Janus particles (FA-SJNCs-DOX) were more toxic reducing the viability of breast cancer cells with an IC_50_ of 255.3 ± 55.1 μg/mL. Moreover, NPs were equipped with multimodal imaging and hyperthermia induced by Fe_3_O_4_.	[162]
PCL-AuNC/Fe(OH)_3_-PAA Janus nanoparticle	doxorubicin (DOX) and docetaxel (DOC)	The loading capacities of docetaxel (DOC) and DOX in PCL-AuNC/Fe(OH)3-PAA JNPs were about 5 wt. % and 20 wt. %, respectively. DOX release rate (60%) at an acidic pH vs. negligible release of DOX in neutral PBS, and it slightly enhanced using NIR laser, while DOC release was enhanced using NIR laser irradiation at 0.5 W cm^−2^ for 5 min up to nearly 60%. The dual-drug-loaded NPs displayed higher cytotoxicity compared with the cocktail (DOX loaded JNPs/DOC-loaded JNPs) and single-drug groups, and CI of 0.42, indicating the synergistic effect of the JNPs, specially in addition to the laser induction.	[165]
UCNP@SiO_2_@mSiO_2_&PMO cubic-spheres	paclitaxel (PTX) and doxorubicin (DOX)	Higher efficiency of cancer cell killing (more than 50%) compared to that of the single drugs (~25%) induced by NIR and UV-Vis irradiations.	[166]
mesoporous silica nanocages	bovine serum albumin (BSA) and doxorubicin (DOX)	Release control capabilities through the specific drug loading site, guest molecules size, and heat and NIR light. The loading capacities were 342 mg/g for BSA and 33.6 mg/g for DOX. Release rates of both DOX and BSA molecules from single-hole nanorattles under the stimuli of heat (up to 40 °C) and NIR light reached nearly 100% after 35 h.	[167]
asymmetric lollipop-like Fe_3_O_4_@SiO_2_&EPMO NPs	curcumin (CUR) and gentamicin (GS)	Higher loading capacity of 249 mg/g for GS than 25.8 mg/g for Cur IN Fe_3_O_4_@SiO_2_&EPMO NPs due to intermolecular hydrogen bonds formed between the OH and amino groups of GS and the silanols (Si−OH) of the hydrophilic mesoporous SiO_2_. The total GS released amount were 99.2 and 96.8%, while 63.0 and 50.3% for DOX from Fe_3_O_4_@SiO_2_-GS&EPMO and Fe_3_O_4_@SiO_2_-GS&EPMO-Cur, respectively. while only Cur and GS kill 48.2% and 19% of cells, respectively, Fe_3_O_4_@SiO_2_-GS&EPMO-Cur kill 89.6% of the cells, indicating the synergic effect of the dual-drug loaded NPs.	[168]
multicompartment hydrogel (MCH)	doxorubicin (DOX) and paclitaxel (PTX)	The drug loading amount was approximately 0.75% (*w/w*) for PTX and 4.2% (*w/w*) for DOX. PTX and DOX accumulative released amount from single-loaded MCH was 62.2% and 20.7%, respectively, while the data was around 64.9% for PTX and 23% for DOX, indicating that drugs were well segregated. MCH loaded with both drugs also had the lowest MCF-7 cell viability at only 10%, lowest tumor volume, and side effects compared to other groups, especially single-drug samples.	[179]
PNIPAM-b-PNAM-b-PNBOC self-assembled hydrogels	gemcitabine (GCT) and doxorubicin (DOX)	Enhanced internalization, and release control and gel-to-sol transition vis UV light and NIR irradiation was observed. The loading efficiencies (LE) were calculated to be 80.5% for DOX and 100.0% for GCT. At 15 wt. % and 37 °C, less than 20% of GCT and 10% of DOX were released without UV, while more than 98% of GCT and nearly 80% of DOX were released with UV inductions, after 70 h.	[180]
PLGA/PLA nano discs	levodopa (LD) and carbidopa (CD)	Sustained release rate for both drugs in acidic conditions. The encapsulation efficiencies were 97% for LD and 78% for CD in system B (LD in PLGA and CD in PLA). The release profiles of system B illustrated nearly 90% of drugs were released after 24 h.	[187]
MSN Janus NPs	doxorubicin and berberine (BER)	Loading efficiencies were 58.81% for DOX and 54.17% for BER. the highest apoptotic efficiency of HA-MSN@DB at 48.10% among other systems, and a considerable growth prevention and tumor proliferation inhibition as well as enhanced cell internalization via CD44 receptor-mediated pathways, were observed.	[196]
H-ZIF-8/polydopamine Janus nanoparticles	doxorubicin hydrochloride (DOX.HCl) and 10-Hydroxycamptothecin (HCPT)	Loading capacity (LC) of H-ZIF-8/PDA-CD JNPs was 42.0 wt. % for DOX and 9.8 wt. % for HCPT. All release profiles at various pH conditions were enhanced after using a laser, where the release rate of DOX and HCPT reached nearly 80% and 60% at pH 5.3. cell viabilities and tumor volumes treated with dual-loaded JNPs plus laser were lower compared to other groups, highlighting the effective PTT and chemotherapy synergy.	[197]
self-assembled hyaluronic acid (HA) and cationic amphipathic starch (CSaSt) NPs	docetaxel (DOC) and doxorubicin (DOX)	The encapsulation efficiencies were 96.1 ± 2.3% for DOC and 91.4 ± 3.7% for DOX. approximately 70% of DOX was released within 12 h when HAase was added compared to 40% without HAase. DDC NPs attenuated the toxicity of DOC and DOX in vivo, with 20% vs. 80% mortality, and lower tumor growth in mice treated with the combined drug treatment. DDC NPs could also enhance the accumulation of drugs in tumors via HA-targeting agents.	[200]
chitosan/sodium alginate/PLGA Janus NPs	venom protein (αCT) and resveratrol (Res)	Improved bioavailability, cell entrance, and intestinal absorption. The entrapment efficiency (EE%) was 58.14% for αCT and 87.49% for Res. Compared with free αCT and Res, the JNPs have obvious sustained-release characteristics where the cumulative release of αCT in αCT-JNP and αCT/Res-JNP was 78.44% and 73.82%, respectively, compared to the cumulative release of 94.41% for Res in Res-JNP and 94.57% from αCT/ResJNP, respectively.	[203]
PVP/PCL Janus nanofibers	Rana chensinensis skin peptides (RCSPs) and Ag NPs	Good wettability, mechanical, antibacterial, Biocompatibility, and wound healing properties. The cumulative release rate of RCSPs from RCSPs-Ag nanofibers reached 94.15% after 35 min. Lowest cell viability and highest wound closure for RCSPs-Ag nanofibers after 7 and 14 days, respectively.	[206]
assembled triblock terpolymers into multicompartment patchy NPs	doxorubicin (DOX) and Cyanine5 (Cy5)	FRET real-time monitoring capacity, simultaneous release and delivery to cancerous cells. The loading efficiencies of DOX and Cy5 were 49.27% and 18.76%, while the co-loading method decreased DOX loading efficiency and capacity by ~15% with negligible changes in the data of Cy5.	[207]

## Data Availability

Data sharing not applicable.
